# Patient data ownership: who owns your health?

**DOI:** 10.1093/jlb/lsab023

**Published:** 2021-10-01

**Authors:** Kathleen Liddell, David A Simon, Anneke Lucassen

**Keywords:** property, ownership, health, information and data, consent, digital health

## Abstract

This article answers two questions from the perspective of United Kingdom law and policy: (i) is health information property? and (ii) should it be? We argue that special features of health information make it unsuitable for conferral of property rights without an extensive system of data-specific rules, like those that govern intellectual property. Additionally, we argue that even if an extensive set of rules were developed, the advantages of a property framework to govern health information would be slight: propertization is unlikely to enhance patient self-determination, increase market efficiency, provide patients a foothold in the data economy, clarify legal uses of information, or encourage data-driven innovation. The better approach is to rely less, not more, on property. We recommend a regulatory model with four signature features: (i) substantial protection for personal health data similar to the GDPR with transparent limits on how, when, and by whom patient data can be accessed, used, and transmitted; (ii) input from relevant stakeholders; (iii) interoperability; and (iv) greater research into a health-data service, rather than goods, model.

## I. INTRODUCTION

### I.A. Background

The big-data revolution in healthcare is well underway. Data-aggregating initiatives in the healthcare sector include electronic health care records created by public and private healthcare providers, biobank collections, plus information generated by medical devices, social media,^1^ and data platform companies. Driven by desires to solve complex health questions, predict new issues, decrease public costs, and increase returns, data science is growing faster in healthcare than other leading market sectors (with a compound annual growth rate of 36 per cent through 2025).^2^ The global value of health data is also growing and expected to reach $34.27 billion by 2022 with a compound annual growth rate of 22 per cent.^3^ In the public sector, the NHS has access to one of the largest single resources of patient data globally, giving the UK a unique opportunity to capitalize on the deployment of data and data-driven technologies in healthcare.^4^ But who owns the patient data held by and generated by these organizations? And does it matter?

With the data economy booming, calls for patients and health services to ‘own’ or wrest control of ‘their’ data are becoming more insistent. These calls are often articulated as a right of ownership or property.^5^ Driving these demands are two overlapping concerns: how organizations handle and use health data and how those organization monetize them as digital assets.

Outcries over data misuse by organizations that claim to be trustworthy are legion. For example, the Royal Free Hospital in Hampstead, London, granted access to 1.6 million health records to Google’s AI subsidiary, DeepMind, to help the company develop an app that analyses test result data for patients in danger of developing acute kidney injury.^6^ Other high-profile UK health data controversies include a weakness in GPs’ IT platforms, which put the records of 26 million patients at risk of being shared with strangers;^7^ an NHS sexual health clinic mistakenly releasing HIV status data on 781 of its patients;^8^ a ransomware cyberattack that shut down NHS computers across the country; and a COVID-19 health data storage deal where private tech companies charged the NHS a mere £1 for services rendered but acquired the ability to test and develop emerging artificial intelligence models on NHS data.^9^ Similar incidents have occurred the world over.^10^ A notable example is the 2019 US-based class-action lawsuit against the University of Chicago and Google, which alleged that the medical center shared hundreds of thousands of patients’ records with the technology giant without adequately removing identifiable date stamps or doctor’s notes.^11^ These incidents generate uncertainty and concern about the security of health data and whether patient data are properly protected. Even where lawyers are not involved, consequences can be serious. One reason the UK government scrapped the NHS’ flagship data (care.data) not long after introducing it, for instance, was because English general practitioners (‘GPs’) did not support it. ^12^

In response, policymakers have acknowledged that data science platforms cannot afford to be merely ‘technical’. They also need to account for legal and ethical issues that surround data development and use. Here, several governance questions emerge regarding the use, sharing, and trading of data (a term we use interchangeably with information^13^): when are these activities permitted, required, recommended, and prohibited? Data science platforms cannot afford to be merely ‘technical’, but need also to account for legal and ethical issues that surround data development and use.

Many think that these questions can be answered by turning to a property analysis of health data—by asking and answering the question, ‘Who owns the information?’^14^ And, perhaps unsurprisingly, many—the Secretary of State for Health, hospital patients, NHS Trusts, medical and research professionals, pharmaceutical and diagnostic companies, biobanks, etc.—claim they own it.^15^ Others argue that the law is clear: information, as a legal matter, cannot be the subject of property. Therefore, no one owns health data.^16^

The aim of this article is to analyze this question and whether patient’s should have property rights in their health data. It does this in five steps. First, it assesses what people mean when they ask about and assert ownership. Second, it investigates whether English law recognizes property in health data, including whether and when patients can own their health data. The inquiry focuses first on property in health data *per se*, and then health data embedded in intellectual property. The conclusion is briefly compared with other legal systems including the United States of America (USA). Third, the article analyses existing non-proprietary protection of patient data in English law. Fourth, it asks whether the law ought to recognize property in data. Finally, it recommends a different way of thinking about and managing patient data and the legal and ethical issues it raises.

The article draws together a wide literature emanating from both sides of the Atlantic, across several decades, and covering both health information and genetic data. While this literature contains both a rich and diverse set of viewpoints, it does not offer a detailed and systematic account legal and policy implications of the issues, particularly in the UK. This article does so. Our paradigm example is the UK law, which complements mostly the US accounts in the literature. But the position we ultimately argue for is not specific to any jurisdiction.

### I.B. Defining health data and health information

For purposes of this article, we define patient health data as:

(1) any and all data generated, created, or collected and retained;
(2) in any form or medium;
(3) by the National Health Service (NHS);
(4) relating to an individual patient;
(5) in, during, or as part of a clinical or clinical research encounter.^17^

This definition is broad, but not exhaustive of data that could reasonably be considered patient data.^18^ It includes, for example, the data typically stored in electronic healthcare and clinical research records, as well as human genetic data. It excludes, however, data generated by patients or third parties outside of clinical encounters with the NHS. Wearable technology (such as the Apple Watch or Fitbit) and direct-to-consumer health care products (such as 23andMe and Ancestry.com third-party genetic testing) also fall outside the scope of this definition. While important, contracts with private healthcare providers and companies raise legal questions beyond the scope of this article.^19^

Although philosophers consider data and information to be distinct concepts,^20^ the law generally does not treat them that way. The European General Data Protection Regulation (‘GDPR’), for example, defines ‘personal data’ as ‘any information relating to an identified…natural person’. This article follows this conflated conceptual definition—treating data and information as synonymous—for two reasons. First, it fits with the article’s aim to assess the existing legal status of patient information. Second, the distinctions that matter for legal rules are principally the differences between kinds of information, and not between data and information.

### I.C. Special features of health data and health information

Patient health information has several features that complicate the question of whether the law should recognize patients as owners of property in some or any of their health information.^21^ First, health information can have both a clinical and personal valence. A person’s ‘height’ and ‘weight’, for example, describe basic patient characteristics. From a clinician’s perspective, these seemingly innocuous data can sometimes be relevant in the diagnoses of risk factors or health conditions such as diabetes and heart disease, as well as refining predictions about current or future health. Collection of height and weight information is a standard feature of many healthcare consultations, and the healthcare provider may treat it as their private data. From a patient’s perspective, however, this information may seem personal and private. While a provider might experience financial loss if data are disclosed, the same disclosure may cause a patient embarrassment, shame, or stigma when that information is disclosed in a public or semi-public fashion.

Second, the value of health information for treatment varies widely depending on both the information itself and how and by whom it is accessed. Some information may be entirely meaningless to both the clinician and the patient, and some may have high relevance to healthcare. An entire genome sequence for an individual, for example, might contain a mutation or copy number variant that is diagnostic or highly predictive (eg, a pathogenic BRCA1 gene mutation, which increases risk of breast and ovarian cancer). But it will also contain a large amount of information that has no known value: nearly 100,000 genetic nucleotides in any genome have no clear known health implications. Over time, the meaning may change as scientific knowledge develops. Variants with unknown significance can become meaningful if knowledge of a clinical association emerges. Complicating matters is that any given patient’s genome may not be valuable for population-based studies, but its inclusion is required for those studies to take place. And patients increasingly understand that participation or inclusion of their data can assist research.

A third issue is that a patient’s health data may include information—including deeply personal information—about third parties. During a routine visit to the doctor, for instance, the healthcare professional may solicit and record a patient’s family history or information about other non-family members. This might include sensitive information about the mental health, genetic conditions, and medical diagnoses. Furthermore, a patient’s health information could have significant implications for other people. A patient who discovers that they have inherited a particular genetic condition might want to protect that information from disclosure. At the same time, failing to disclose that information could pose a health risk, or at least impede decision-making about a health risk, to a family member who may also have or carry the disease. Similarly, a patient diagnosed with a sexually transmitted disease might consider this private and potentially stigmatizing information. But sexual partners may have an interest both in knowing their potential exposure and in limiting exposure to others. With respect to the former, knowledge can enable treatment. With respect to the latter, knowledge can enable behavior that prevents or reduces risk that others will be infected.

Fourth, health information usually is not generated solely or predominantly by the patient; rather, it is constructed by number of different parties and devices.^22^ Consider an innocuous trip to the clinician. A presenting patient might describe symptoms of wound that will not heal. The clinician’s subsequent investigation of these data—by, for example, examining the patient and ordering laboratory tests—creates more data, which is processed and interpreted before being documented or reported to the patient. Health information in this context covers a wide range of data, including the patient’s account of how the injury occurred; the self-described symptoms; the data acquired through investigation of that presenting complaint (examination, raw data, and measurements); the clinician’s interpretation of the patient’s description, as well as their subsequent notes, observations, and diagnoses; additional data acquired by the health service providers; and other information stored in the patient’s electronic health record. Health information, in other words, arises and is created through complex processes that involve bodies, perception, interpretation, measurement, work, skill, judgment, and equipment. Both the patient and the professional are necessary, but not sufficient, to generate health information. Without the patient, there is no information at all; but without professionals there is negligible or no health information.

The foregoing issues highlight significant questions about the value, meaning, and origins of health information. Its value and meaning can vary with perspective, context, and time. It would be over-inclusive to assume that all health information is sensitive, clinically useful, or economically valuable. As noted above, it would also be too limiting to assume that the individual is the only source of information about herself. An individual’s health information could emanate from that individual or from another person (eg family history, shared health, and genetic information). It may also be publicly available (eg visible characteristics, such as skin color), semi-public (eg weight and height), or private (eg genetic data). And in clinical settings, health information is typically co-constructed with healthcare providers and is rarely provided solely by the patient.

We discuss the implications of these features for ownership and a property framework later in this article. In general, we show that these features make it challenging to propertize health information in any useful way. Health information, for example, is difficult to possess exclusively. Natural inferences of copying also pose a challenge: the fact that information appears in more than one context may mean that it is recorded in those different contexts, rather than appropriated from one to the other. For the same reason, devising and dividing rights based on concepts like ‘first possession’ and ‘allocation’ present further challenges to using property to protect health information. How can interests in health information be extinguished when the information itself may arise naturally or be created in another context—or when events occur that influence whether the data are considered health data at all? Finally, definitional problems and data operations, such as pseudonymization or full anonymization, plague a coherent and stable property framework. Since the precise scope of health information can shift rapidly, propertizing ‘health data’ may inadvertently propertize data that no longer links to the recognized ‘owner’, is no longer created by the initial creator, or no longer concerns health. For instance, a different medical perspective, an addition (another blood test), or linkage to another database can lead rapidly to additional health information or different data diagnosis or prognosis. We explore these problems in more detail below.

## II. LANGUAGES OF OWNERSHIP

### II.A. Assertions of ownership by non-lawyers

Refrains such as ‘the hospital owns the data’ or ‘it’s my health data’—are very common.^23^ They are driven, at least in part, by assumptions made by providers, bioinformaticians, and patients. Sometimes health professionals rely on the assertion because they think it will help them decide or justify what they can lawfully do with the information.^24^ Sometimes hospitals with more enterprising dispositions assume that, as an owner, they have rights—or even responsibilities—to generate value from the information. In these situations, ownership is a way to monetize or capture value that organizations or clinicians feel they have ‘created’.

Patients, too, have ownership instincts; but they are not always of the enterprising variety. Many feel that ‘personal information’ should be owned, circularly, because it is personal. For instance, after giving evidence exposing Cambridge Analytica’s data harvesting practices, Brittany Kaiser, former Business Director at the company, became a public speaker with the handle #OwnYourOwnData. Kaiser started a petition in 2018 with the strap line, ‘Mark Zuckerberg, change Facebook’s rules and give us back control over our data, our digital assets, our property’.^25^ At the time of writing (December 7, 2020) there were more than 179,000 signatures. Similarly, Eric Topol, an influential international leader of physicians and researchers leading the US precision medicine initiative, has asserted that patient ownership of data should be treated as a civil right.^26^ Former President Barack Obama added in relation to the same initiative, ‘I would like to think that if somebody does a test on me or my genes, that that’s mine’.^27^

Organizations are also offering individuals platforms to exchange and monetize their data. Some use the language of ‘data trusts’. For instance, following the 2017 Independent Review of Artificial Intelligence for the UK government, the Open Data Institute joined forces with the Office for Artificial Intelligence and Innovate UK to assess data trusts^28^ as a potential approach to increasing trust and access to data. Some authors have specifically proposed that data trusts be used for medical data.^29^ Others opt for the language of ‘banking’; one such example is ‘healthbank’ in Switzerland that offers to revolutionize ‘how personal healthcare data are exchanged, stored, and monetized’.^30^

Medical professionals, universities, pharmaceutical companies, biobanks, clinical research organizations, and app developers also claim ownership. For example, a number of large human biobanks, which invest millions in collection, assimilation, curation, and storage of data, have provisions in their policies asserting ownership over their collections. The UK’s flagship 100,000 Genomes Project states that “the Project Data [defined as ‘any data created or derived in the course of carrying out the Project including genome sequences and clinical data from Participants’]…should always remain publicly owned and controlled’.^31^ Similarly, Biobank Sverige claims ‘all rights to coded personal data’, and Generation Scotland’s policies state that ‘data/materials remain the property of some or all of the Generation Scotland Collaborating Parties’.^32^ Similar policies can be found with the US Million Women’s Study, the Australian Prostate Cancer BioResource, the Australian Breast Cancer Tissue Bank, China Kadoorie Biobank, the Latvian Genome Database, the IARC Biobank, the Karolinska Institute biobank, the Indian Sapien Biosciences resource and the THL Biobank.^33^ Technology and medical device companies typically include similar clauses in data license agreements.^34^ For example 23andMe’s terms of service for its direct-to-consumer ancestry genetic testing reportedly included a ‘Waiver of Property Rights’ for users of its service in 2018.^35^

Despite these myriad claims to health data, it is just as common to hear doubts about who owns it.^36^ Ballantyne argues that the language of ownership is a metaphor for assertions and doubts about the control and management of health data.^37^ Citizens are concerned about potential disenfranchisement, organizations seek the power to reap benefits from their work with data, and health professionals try to follow appropriate principles of data management (of which ownership could be one). In other words, the language of ownership is a statement about a legal or natural person’s relationship to the data. Ownership becomes synonymous with a strong connection.

Ballantyne points out that a strong connection need not be discussed in terms of ownership. One can talk about ‘my child’ or ‘my University’ without asserting ownership over the child or University—ownership is not necessary in order to assert rights of control and empowerment. Moreover, in her view, ownership is problematic because it is associated with property rights. She argues that people mistakenly think that property rights will enhance and protect their connection with their data which, as we will see, is not always the case.^38^

We agree with Ballantyne that, in non-legal circles, assertions and questions about ownership currently work as a metaphor for describing complicated feelings about health information rather than a lodestar for grounded data management decisions. Recent empirical research by Sorbie et al. further supports in this view.^39^ In the remainder of this article, we investigate more deeply whether the law recognizes data ownership and property in information, and whether it should.

### II.B. The meaning of ownership and property in law

From a legal perspective, ownership is the state of having exclusive rights and control over property.^40^ This raises the question, ‘what is property?’ Various conceptions have been described.^41^ Property is often thought of as a relation between an individual and a thing. But property rights create duties and obligations between people to protect the relation between an individual and a thing. For this reason, property frequently is referred to as a ‘bundle’ or ‘collection’ of rights rather than a single right or thing.^42^

One stick in this bundle is the right to exclude.^43^ This feature is central to the property owner’s control and dominion over the thing in question. Excluding others from using the thing might correspond to an exclusive right to use. But it is not necessarily the right to use in any manner. The owner of a piece of land, for instance, has an exclusive right to use the land. Rules relating to land use however, may curtail the owner’s ability to keep livestock or set up a car repair shop. Public health and welfare may even entitle the government to confiscate property.^44^ Indeed, some ‘property rights’, such as those conferred by patent law, do not provide the owner with any right to use the thing but rather only the right to exclude others from using it.^45^

Another stick in the bundle is the ability to convey the thing, or interests in the thing, to another party. This is called alienability.^46^ This feature is central to current thinking, which conceives of property as a means to facilitate trade.^47^ Law recognizes something as property, in other words, not because it has certain features but rather to provide it with the features necessary to trade in the open market. Flexibility in how property is alienated makes it a highly versatile concept—since property is a collection of rights, not all of these rights need be conveyed simultaneously. One can convey or transfer all or part of an interest in property, and for varying durations. For example, one can assign, sell, or lease property or a part of the property. This is called divisibility.

A fourth recognizable feature of property—another stick in the bundle—is ‘absolute enforceability’. Unlike other legal rights, a property right is good against the world. Practically speaking, it means that anyone who interferes with a person’s property rights violates them and can, as a result, be subject to legal action. This contrasts with contract rights, which can be enforced only against those with whom one has a contract (ie those in ‘privity’). If A contracts with B for 50 widgets, A cannot, absent special circumstances, sue C in contract for B’s failure to manufacture them. A could, however, sue any person who unlawfully takes her widgets. In law, this difference is explained as the difference between having rights specific to a thing (*in rem*) versus having rights specific to a person (*in personam*).^48^

The ‘bundle of sticks’ approach is not unqualified, nor uncriticized. The sticks are not universal for all objects of property. Even many well-established subjects of property (eg, houses, cars, patents) vary in the extent to which they have the features of excludability, alienability, divisibility, and absolute enforceability. Different types of objects imply different legal relations, rights, duties, etc. The same is true for different owners: individuals, corporations, companies, trusts, and so on. For some ‘bundle of rights’ property theorists, the fact that some ‘sticks’ are missing is not problematic; for others, the bundle falls apart when even one stick is missing. This variation in attitude led Jeremy Waldron to note, ‘the common word “ownership”—“X owns the car”, ‘Y owns the land’, ‘Z owns the copyright’—may be unhelpful and misleading, for it cannot convey any common content for these quite different bundles of legal relations’.^49^

Identifying property according to conceptual features (eg, ‘the bundle of sticks’) is a difficult task. To combat this problem, some argue for a kind of property ‘nominalism’. On this view, property ‘is’ whatever the legal system decides to call property.^50^ If an authority declares something to be the subject of property or ownership, it is then a further question whether and to what extent it has the typical features of ‘property’. In the next section, we discuss the extent to which the English legal system has been willing—nominally—to confer the status ‘property’ on information.

## III. THE CURRENT LEGAL POSITION: IS HEALTH INFORMATION PROPERTY? WHO OWNS IT?

Does English law recognize a right for a person to have property in and ownership of health information *per se*? This question arises in contracts for data transfer, non-compete agreements, and confidentiality agreements; academic conferences; interdisciplinary workshops; textbooks; and the courtroom. A variety of views persist. In transactional settings—such as the sale of a business or business assets—data are commonly treated as property. In other settings, the typical legal response is that information is not the subject of property.^51^

Like most things that seem simple, however, the answer is actually more complex. In this section, we unpack the complexity. To accomplish this, we first survey areas of law where UK courts have discussed whether information *per se* can be the subject of property, and protected by property law. Second, we survey the field of intellectual property law. We examine how several areas of intellectual property law—copyright, patent, database rights, and trade secret law—protect information as property.

This section serves three purposes. First it shows that persons^52^ can own specific arrangements, forms, or representations of health information through intellectual property law, although English law has not yet established a definitive principle that a person can (or cannot) own information *per se*. Second, it shows that where intellectual property extends protection to health information, the owner of the property will rarely be the patient from whom information was derived. Third, by explaining some of the nuances of intellectual property rights, this section shows that a workable property system for health information would need to mirror the ‘architecture’ of intellectual property frameworks. This includes rules about inherent eligibility, qualifying criteria, scope and duration of protection, fair notice for third parties, and exceptions from liability and remedies. Without these features, a system of property in health information *per se* would collapse from legal uncertainty and confusion.^53^

### III.A. Property in information *per se*

A case frequently cited for the response that information is not and cannot be property under English law is the House of Lords’ decision in *Boardman v Phipps*.^54^ There, Lord Upjohn, proclaimed that, ‘[i]n general, information is not property at all’.^55^ What matters, Lord Upjohn wrote, is whether a party uses the information and that use breaches a confidential relationship. In such cases, ‘equity will restrain its transmission…’.^56^

In fact, though, the case was more nuanced. The remaining Lord Justices were more sympathetic to the view that information can be property, depending on the circumstances and the meaning of the term ‘property’.^57^ Lord Viscount Dilhorne, for example, stated that ‘some information and knowledge may properly be regarded as property….’^58^ Lord Guest agreed, seeing ‘no reason why information and knowledge cannot be trust property’.^59^ Lord Hodson, like the lower courts, was even more supportive, expressly stating that ‘the confidential information acquired in this case which was capable of being, and was, turned to account can be properly regarded as the property of the trust’.^60^

This case, however, should not be read as categorically supporting the principle that information *per se* can (or cannot) be property. Boardman involved defendants who knew valuable information about a company owing to their roles in a family will trust (which owned a minority stake in the company) and used that information to their advantage when they bought a majority share in their private capacity. This amounted to a conflict of interest. The central questions of the case were, first, whether information was property of the trust, and, if so, whether the defendants owed to the plaintiffs profits they made by selling the company.

Given these facts, it is misleading, though understandable, that the case is quoted as one purely about whether ‘information was property’. The trial judge’s statement that ‘knowledge’ of which profitable use was made ‘was essentially the property of the trust’, gives credence to this view.^61^ In fact, though, the case was less about information-as-property, and more about the law of trusts and trustees and ‘accounting for profits’—concepts that are based on principles of equity and fiduciary law.^62^ Although some Lords purported to address these issues by assessing whether the information obtained was ‘property’,^63^ this step in their reasoning was a nominalist one (see above Section II.B) and depended not on a pre-ordained rule about information as ‘property’ but rather how the information was obtained^64^ and ‘the use to which it is applied’.^65^*Boardman v Phipps* is useful, then, not as an authority on whether information *per se* definitively can be property, but as an example of how courts will sometimes resort to the language of property as a proxy for the concept of unlawful misappropriation.

In the 50 years since *Boardman*, no English case has definitely established whether information *per se* can (or cannot) be treated as property. Two relatively recent cases illustrate the difficulties of disentangling the property analysis from other areas of law. The first case is *Fairstar Heavy Transport NV v Adkins*, in which Fairstar sought emails drafted by its former CEO (Adkins), the only copies of which were stored on Adkins’ personal computer (to which Adkins was unwilling to provide access). Fairstar’s lawyers argued that the contents of the email were its property. At first instance, the court rejected this argument and held that there was no property right in information that made up the contents of an e-mail. Consequently, the court rejected the company’s right to inspect and copy the contents of the emails.

The Court of Appeal reversed the lower court’s decision on principles of agency law. It held that the agency relationship between Adkins and Fairstar entitled the company to inspect and copy documents held by Adkins even after the agency relationship ended. However, since agency law dispensed with the issue, the Court of Appeal was non-committal on whether the law protects (email) information as property. It was not prepared to endorse the lower court’s view that information cannot be property.^66^ Mummery LJ, who gave the leading judgment, offered several important asides. He noted that a claim to property in intangible information presented obvious ‘definitional difficulties’. In particular, information did not lend itself to criteria of certainty, exclusivity, control and assignability that normally characterize property rights. However, he added that

[i]t would be unwise…for this court to endorse the proposition that there can never be property in information without knowing more about the nature of the information in dispute and the circumstances in which a property right was being asserted.^67^

The Court of Appeal took a very similar view in the more recent case of *Your Response Ltd v Datateam Business Media Ltd*.^68^ Datateam Business, a database manager, argued that an electronic database was a type of property (either tangible or intangible) capable of possession and thus of being subject to a ‘lien’. The Court of Appeal held that the law of liens extended only to tangible property in the possession of the bailee. While it was noted that the health records (paper- or computer-based) on which the information was contained could be considered tangible pieces of personal property, a database itself was merely intangible intellectual property (see Section III.B.2) and the court was not willing to recognize the information *per se* as tangible personal property. Lord Justice Moore-Bick, giving the leading judgment, expressed limited support for such an extension of property rights but concluded that such a ruling would be inappropriate: ^69^

In my view there is much force in [the appellant’s] analysis, which, if accepted, would have the beneficial effect of extending the protection of property rights in a way that would take account of recent technological developments. However, to take the course which they propose would involve a significant departure from the existing law [on liens] in a way that is inconsistent with the decision in *OBG v Allan*. That course is not open to us—indeed, it may now have to await the intervention of Parliament—and I do not think that any purpose would therefore be served by embarking on a fuller discussion of their suggestions here.^70^

Lord Justice Floyd and Lord Justice Davis agreed and added some concerns in *obiter dicta* about possible unintended consequences.^71^ Lord Justice Floyd noted that the law has been reluctant to treat information itself as property.^72^ In support he cited Lord Walker’s *obiter dictum* in *Douglas v Hello!* that ‘information, even if it is confidential, cannot properly be regarded as a form of property’.^73^ As noted by Alastair Hudson, though, Lord Walker’s ‘unqualified remark did not consider the various judgments in *Boardman v Phipps*. (mentioning only Lord Upjohn)’.^74^

Like the *Boardman* court, the *Fairstar* and *Datateam* courts decided the case based on special circumstances surrounding the use of information: in *Fairstar* the agency relationship; in *Datateam* the law of liens; and in *Boardman* the fiduciary relationship. In all of these cases, the courts left open the possibility that information *per se* might be treated as property, but declined to make it so in each case.

This analysis therefore shows that English law has been reluctant to treat information itself as property,^75^ but has not ruled out this position. English law thus leaves open question the question of whether information can be property; and perhaps the courts might reconsider the issue with information being a prized asset in the modern data-driven economy.

In the next sub-section, we go a step further in our legal analysis and draw attention to the field of intellectual property law. Here the law has developed several sets of principles that confirm that information can indeed be property. So, we conclude, at least within intellectual property law, English law permits ownership of health information.

### III.B. Intellectual property

Intellectual property law is a ‘family’ of somewhat loosely related legal frameworks which provide rights, generally property rights, in qualifying circumstances.^76^ It includes patent law, copyright law, and trade mark law, as well as the European database right. In addition, trade secret protection and confidential information are often treated as being within the intellectual property law framework;^77^ however, there is no consensus on whether trade secrets or confidential information actually constitute property. The prevailing view in the UK is that they do not.^78^ Within intellectual property law there are rules governing the protection, duration, and infringement of rights in information. Intellectual property law also further limits the property rights by stipulating defenses to liability. These limitations and defenses are designed to curb the negative social consequences of granting property rights in information.

#### 1. Copyright and copyright in databases

Certain forms of patient health information are protected under the law of copyright. Copyright protects the ‘original’ ‘intellectual creation’ of an ‘author’ across a range of cultural goods, including books, songs, films, and computer programs.^79^ In the case of written works, the protection lasts 70 years after the author’s death. In the UK, a work is original if it demonstrates the author’s ‘labor, skill or judgment’,^80^ or it is the author’s ‘own intellectual creation’.^81^ The result is that expressions and compilations of facts about patients— such as a doctor’s analysis and notes of a patient’s metabolic levels—may be protected under English copyright law. Even compilations of recorded height, weight, or genetic variation may be protected provided they evince sufficient ‘selection and arrangement’ to ‘constitute the author’s intellectual creation’. Ownership of the physical record, the paper or electronic copy, is treated separately from the intangible information. A similar standard exists in other jurisdictions, including the USA.^82^

UK copyright law does not protect facts or ideas. It does, however, protect expressions of facts and ideas insofar as they constitute the author’s creative intellect, labor, skill, or judgment. This means that doctors may claim copyright over patient information (including facts and diagnostic opinions) if they select, assemble, and arrange it in an original way. But the ownership rights would extend only to the arrangement, not to the underlying information itself.

In contrast to doctors, patients’ claims to copyright would usually be weak. When a patient records her own health information using a health ‘app’, for example, the patient is not selecting or arranging the information she records through their own initiative; she is, in many cases, following prompts from the app, which organize the information for the patient, the same way a doctor or nurse would on a chart. Additionally, a company might also use contractual licensing agreements in its Terms of Service to exclude any claim the user might have to the information recorded or generated by the app.^83^ The same contractual terms may also enable the company to use the patient’s information for its own purposes, including selling it to third-parties.^84^

All considered, copyright law can protect information as property for a lengthy period. Significantly, though, doctrinal rules make it far more likely that health care professionals and organizations, not patients, will own property in patient health information. Moreover, rules governing copyright scope and infringement narrow the range of protected information. For example, copyright cannot be enforced against an individual who independently creates the same or similar information subject to copyright protection. If two physicians independently compile the same information by asking a patient the same questions on separate occasions, each will have a separate claim to the selection and arrangement of the information created. Beyond this, copyright also permits third-parties to express facts and ideas, which are not subject to copyright, however they see fit. Although copying an entire health record could breach copyright, unauthorized re-use of discrete units of health information *per se* (many of which are likely to be ‘facts’) is unlikely to amount to an enforceable copyright infringement. To be actionable, the user would have to appropriate a sufficient quantity of information representative of the original effort/creativity in making the record. This provides an internal check on a property system without registration requirements. Although one acquires copyright without formal registration, the internal mechanisms of copyright law place a user on notice that they might be infringing copyright property whenever they engage in an act of substantial copying. Additional certainty flows from the requirement that (most) works must be ‘fixed’ or recorded.^85^ Even when the user copies, several defenses to liability also exist, including research and private study. Special schemes exist to license copyright works if their owner cannot be identified.

#### 2. Database rights under EU Law

Patient information might also be considered property under the special protection conferred by the EU Database Directive. This law protects databases in which an investment has been made.^86^ In this context, ‘database’ means ‘information’ of almost any kind that is ‘(a)…arranged in a systematic or methodical way, and (b)…individually accessible by electronic or other means’.^87^ To gain protection of a database in the UK, there must be a ‘substantial investment in obtaining, verifying[,] or presenting the contents of the database’.^88^ The legislation stipulates that the database right is a property right.^89^ The right, which lasts 15 years (but this recommences if there is another substantial investment such as updating),^90^ gives the owner the legal right to prevent use of the whole or a substantial part (evaluated by qualitative and/or quantitative analysis) of the contents of the database. The owner also has the legal right to prevent repeated and systematic use of insubstantial parts of the contents of the database.^91^ A database made available to the public may be used for scientific research without explicit permission.^92^

The first owner of the protected database is the person who ‘takes the initiative and the risk of investing’.^93^ The ‘investment’ consists in seeking out existing independent materials, collecting them, and making them accessible (eg, sortable, usable). So it is not enough that there was investment in the creation of the data that is the subject of the database.^94^ Investment in a clinical trial, for example, does not result in the trial data being protected. If, however, the trial investigators organized, maintained, and presented the trial data clearly and intelligibly, they would be entitled to database protection (as is the case, for example, for electronic health record systems, biobanks, and genomic databases^95^). So while it is possible for organizations that collect patient data to obtain property rights in databases, it is much more difficult for individual patients to obtain database rights (a property right) in their own health information. To do so, they would have to collect it in a systematic way and make it accessible.

#### 3. Patent law

Patent law also grants property in, and ownership of, information—at least information describing a new, non-obvious and useful inventive concept. Section 30(1) of the Patents Act 1977 (UK) states that patents are a form of property.^96^ Patented methods—which cover processes rather than products—are clear examples of property in information because a method never has any physical form. Patents for inventive products are also, in a way, informational property since what patent law protects is the information described in the form of information (‘claims’) at the end of the patent document—not any physical object.^97^ As stipulated in the European Patent Convention, these ‘claims’ determine the scope of protection conferred by the patent. In other words, the claims determine the boundaries of the property.^98^

To qualify for protection, a patent claim must pertain to an eligible invention, that is new, non-obvious (ie, involve an inventive step), susceptible of at least one useful application, and sufficiently disclosed.^99^ It must not extend over material that was previously made available to the public or that was obvious. Patient information could be the basis for a method or product claim, but only if the inventor, who is likely to be medically or scientifically trained, combines it with an inventive step. Significantly, the owner of the patent property is the inventor or inventors who provided the creative heart of the invention, not the person who provided information or resources.^100^ This means that patients are unlikely to own any patent, even though their health information may have been essential to the realization of the invention. For instance, if a group of patients report to a doctor that they suffered some nausea but their eczema has been much less painful since they entered a clinical trial for a medicine for their heart condition, the patients would have no rights to a patent that covered the new use of the medicine (for the treatment of eczema).

This last example illustrates that patent law confers property over not just information about physical objects, but also to new medical uses for known substances and compositions.^101^ Although these patent claims formally take the form of product-use rights, the protection in effect covers the new use information^102^ when used to treat a human or animal body. The medical product is largely unaffected. It can be used and even patented again for other purposes. European law has also been generous towards the patenting of genetic information. The ‘isolated’ sequence or partial sequence of a gene is eligible for patent protection, even if it is identical to the sequence in a natural un-isolated gene and even if the value rests in the sequence information rather than the physical structure of the nucleotides.^103^

Patent protection is broad but also carefully limited. One limit is time. Patent protection typically lasts up to 20 years from the date of priority, which is often the date of filing. Other limitations are the actual rights patent law allows the owner to assert against others. To sue for infringement of the patented property, the owner must show that a third party, acting with the owner’s consent, (i) makes, sells, offers to sell, uses, imports or stores the production where the invention is a product;^104^ or (ii) uses the process or offers it for use, or he sells, offers to sell, uses, imports or sells any product obtained directly by means of that process where the invention is a process.^105^ In both cases, the allegedly infringing variant must fall within the normal interpretation of at least one of the patent claims, or vary in an immaterial way.^106^

Finally, as with copyright and database rights, infringement may be excused if the defendant has a legal defense. UK law, for example, provides a defense for using protected products and methods for the purposes of research on the product or method.^107^ This allows researchers to investigate the properties of patented products, or to improve the invention, even when their research is commercially motivated. However, once their activity is no longer geared towards answering research questions, the defense for research purposes is inapplicable.

#### 4. Confidential information, trade secrets, and breach of confidence

The law of confidence and the law of trade secrets also (arguably) belong to the intellectual property ‘stable’.^108^ These areas of law are largely coterminous.^109^ The main difference between them is the set of criteria required for protection. ‘Confidential information’ can include almost any kind of information conveyed in confidence. A ‘trade secret’, on the other hand, is information that is commercially valuable because of its secrecy, is not generally known or accessible, and is kept secret by its possessor taking reasonable steps to limit its disclosure.^110^ In this article, we limit our discussion to confidential information for two reasons. First, there exists under the Directive a substantial body of case law on confidential information but not trade secret protection. Second, patient data are typically confidential but not necessary a trade secret.

The law provides protection for confidential information *per se* (including patient data), but it is not clear whether such protection constitutes ‘property’ in the UK.^111^ Though some cases seemingly support treating confidential information as property,^112^ Aplin, for one, argues that the weight of opinion and case law tips in the other direction (not property). She also takes the view that even in the cases supporting confidential-information-as-property, judicial support is hedged. For instance, the cases use ‘property’ language metaphorically or as an analogy to import copyright concepts, or adopt property concepts, in an effort to find liability against third party strangers.^113^

It is also doubtful whether confidential information satisfies another central attribute of property: alienability. Bently et al. note that there are at least six different views on whether and in what circumstances confidential information can be alienated, including conflicting authority about whether it can be assigned. One line of cases suggests that while confidential information is not assignable, the benefit of confidentiality is.^114^ So although one might transfer the obligation to keep the information secret, the obligation is good only against the party to whom it applies. In this respect it is unlike property because it is not ‘good against the whole world’.^115^

Whatever the answer to the property question, the law of confidential information, like other areas of intellectual property described above, sets limits on the law’s protection. Unlike copyright and patent law, however, the law of confidential information protects any information provided that the information ‘has the necessary quality of confidence’ about it,^116^ is ‘imparted in circumstances importing an obligation of confidence’,^117^ and is used without authorization in a way that causes damage to the person who conveyed the information in confidence. Certain relationships, for example also import an obligation of confidentiality, including the doctor-patient relationship.^118^ As a result, courts determining whether an individual violates the law of confidence focus on how third parties use information, or the circumstances under which they do so. This emphasis also explains why these concerns—and not whether the information, as such, can be owned as property—tend to dominate courts’ analysis (as seen in *Boardman* and *Adkins*^119^). The reason for the courts focusing on legitimate circumstances of use is that the law of confidentiality is linked to a duty of good faith or a reasonable expectation of privacy.

Health information is typically considered the subject of personal confidentiality,^120^ since it is often disclosed in circumstances importing a duty of confidentiality (eg, during a clinical consultation) or associated with a reasonable expectation of privacy. The duty of confidentiality and the duty to respect privacy is often owed to the patient, but can also be owed to other people such as family members if they too have a reasonable expectation of privacy. Doctors are not the only ones who owe duties of confidentiality to these individuals. Any other person who acquires information under a duty of confidentiality or handles it in circumstances where the subject has a reasonable expectation of privacy is under a duty not to misuse it (eg, computer technicians, auditors, and researchers).

If personal information such as health data can form the basis for an action for a breach of confidence claim, when does one breach the duty to keep such information confidential? This question can arise with respect to anonymized and pseudonymized data (leaving aside the question of whether and when ‘anonymization’ is still possible in the era of big data). With respect to anonymized personal health information, at least one court has found that selling such information once it is anonymized does not violate the law of confidence. The Court of Appeal in *R. v Source Informatics Limited* found that an arrangement whereby the defendant obtained anonymized patient information from pharmacists did not mean that pharmacists breached their duty of confidentiality. The court asked whether ‘a reasonable pharmacist’s conscience [would] be troubled’ by such a scheme, and held that anonymization meant it would not. The patient’s case could not be rescued by the patient claiming to have property in the information since the ‘patient has no proprietorial claim to the prescription form or to the information it contains’.

### III.C. An emergent framework for recognizing property

Before recognizing a new category of property, such as health information, Professors Bridges et al., authors of the seminal UK text on personal property, The Law of Personal Property, offer clear and simple guidance.^121^ They quote Lord Wilberforce’s opinion in *National Provincial Bank Ltd v Ainsworth* (when deciding to reject the submission that a deserted wife’s share of the former matrimonial home is a form of property right):

Before a right or an interest can be admitted into the category of property, or of a right affecting property, it must be definable, identifiable by third parties, capable in its nature of assumption by third parties, and have some degree of permanence or stability.^122^

Professors Aplin et al. in the major UK text on breach of confidence—where debates about the nature of property status are rife—suggest a similar list of pre-requisites for the recognition of property:

(1) the thing to be recognized as property needs to be easily defined or demarcated;
(2) it must be practical to recognize exclusivity in the thing either by force of law or by its nature;
(3) it must be practical to apply notions of first and subsequent ownership (for instance, initial ‘generation’ of information can be attributed to particular person(s)); and
(4) it must be practical to control when and if the thing is shared—if the thing is too easily shared and other people are not on notice that the thing is the property of someone else, it would be problematic to recognize it as property.

The collective wisdom from these scholars also echoes lessons that can be drawn from the legal organization of intellectual property law—an entire field devoted to conferring property rights in certain types of information. As noted, while copyright, database rights, and patents each extend property rights over information, all stop short of property rights in information *per se*. They do so for important reasons (eg, to protect the public domain, to encourage innovation and creativity) and with sophisticated rules (eg, rules about inherent eligibility, qualifying criteria, scope and duration of protection, fair notice for third parties, and exceptions from liability for infringement and remedies). Many of intellectual property’s rules give information the attributes referred to by Bridges et al., and Aplin et al.: sufficiently precise boundaries, fair notice to third parties, stability, and notions of first and subsequent ownership. They also demonstrate that carefully circumscribed limits to protection (duration and defenses to liability) are important fail-safes against the social harms that might follow from protecting information to broadly. For example, rights that covered inventions that lacked utility or an inventive step would threaten to collapse patent property under the weight of trivialities.

Each intellectual property regime carefully balances the costs of protecting information against the benefits of doing so in limited ways. And each has its own doctrinal tools for doing so.^123^ Copyright, for example, limits protection to an original work based on a person’s intellectual creation or labor, skill and judgment. For database rights, protection is limited to a database which involved substantial investment to organize. For patents it is a new, non-obvious invention. The person who imparts the central qualifying feature (ie, originality, investment, or inventive concept) is recognized as the first person entitled to the property, and all subsequent claims must be linked to them through a trail of licensing or assignment.

A third-party crosses the legal boundaries of copyright, database and patent protection when they use the work, database, or invention in a way prohibited by law—for example, by copying the work (for copyright and database property) or by making the invention (in patent). This is a major restriction of liberty. But the law tolerates it generally in view of the countervailing advantages, and third parties are given notice, albeit in different ways. In patent law, patents are registered with a central office and published for inspection by anyone who cares to look. With copyright and database protection, formal registration is not an option in the UK^124^, but users are only liable if they copy a substantial part, or in the case of database rights repeatedly copy insubstantial parts. This, coupled with requirements to sufficiently acknowledge a copyright author, means that a user has a degree of notice before they infringe these property rights. The duration of each type of right is again tuned to the different goals of each legal regime (patents are much shorter), but importantly neither copyright nor database rights last indefinitely. And both patent and copyright also have extensive provisions (in the form of defenses to liability) that balance the legitimate claims non-authors/non-inventors to use the work/invention and associated information for socially beneficial purposes (eg, research, teaching, follow-on innovation and creativity).^125^

Health information, if recognized as property, contains no such independent, highly-tailored rules. This raises challenges both to recognizing it as property and to managing it as such. In particular, a lack of subject-specific rules means there are real social risks to recognizing health information as property. And some of these risks flow directly from the inability to characterize health information as property. As explained in Section I.C, health information is difficult to define and demarcate. There are no reliable bright lines that distinguish health and non-health information, precursors to information and information, or information about one person from information about a relative. Contrived definitions are possible, but they will be vague. And without qualifying criteria, the lack of definition will remain.^126^

Furthermore, it is not practical to apply notions of first and subsequent ownership to health information, especially in fluid data ecosystems and networked IT systems, or in the absence of criteria for demarcating stable property boundaries.^127^ A patient might stake a claim based on the notion that the information is ‘about’ them; but medical personnel might also stake an overlapping claim for the information that they ‘generated’ through their involvement. Subsequent ownership claims could then become very confused, especially if the patient and professionals seek to divide the property—licensing aspects of it to different people. Information also is insufficiently stable if it is demarcated by the most intuitive feature of health information: whether it identifies a particular person. And this kind of identifiability can change depending on whether certain details are erased, or other data are obtained and linked. Relatedly, health information does not tend to rest stably in one form or with one source. It can be read, independently generated, or acquired through all sorts of various other routes (eg, through family members, personal observation, deduction from all sorts of indicators). This makes it difficult to maintain exclusivity, either practically or by law.

All these difficulties with recognizing property in health information converge to create two problems. The first is that third parties have very little notice of when health information is property. Requiring copying, fixation and public, notice (eg, through publication or registration) as a precondition of property in health information could ameliorate this concern, but it would likely push out less-sophisticated parties—the majority of patients. This precise challenge animates other areas of law that lack proper notice functions. One of the difficult questions in the law of confidential information, for example, is how to evaluate the position of parties who use information in good faith without notice, believing it was unencumbered. The second problem is the imbalance between the first owner (whoever that is) and any subsequent users or owners. Experience with intellectual property law shows that even a finely-tuned regime has difficulty balancing the rights of owners and subsequent legitimate users.^128^ This does not bode well for a potential property in something, like health information, that is quite difficult to define and where legitimate uses might include public health, medical research, and management of social care systems.

Because of these difficulties, crafting a new property regime for health information may reduce claims of property in health information to merely claims of intellectual property law. Professor Contreras has considered a related question: can features of intellectual property law be transplanted into a system for property in health information to avoid sweeping and perpetual rights? He notes the enormity of the task and questions whether the result would ultimately be a system of governmental regulation rather than one of property.^129^ We share his concern that the task of hewing a property right in health information would be extensive.^130^ And, for similar reasons, we urge a regulatory approach rather than a propertized one.

### III.D. Foreign law

The current legal position in the UK is similar to that of other Western European countries. Neither France nor Germany, for example, have an established law or principle that information can (or cannot) be owned as property (outside of intellectual property rights). While German courts have been relatively clear that data subjects do not own property in data, the French courts have been less so. In both jurisdictions, occasional cases suggest it may be possible that the law might develop towards a proprietary framework.^131^ For instance, the French Supreme Court (*Cour de cassation*) stated that the offence of theft may arise if computer data are downloaded remotely without taking away the computer hardware. According to some commentators, this ruling opens the way to a general recognition of the theft of information by treating information as an object of property.^132^ Similarly, the Federal Supreme Court of Germany (*Bundesgerichtshof*) ruled that software could be the subject of ownership, and that the loss of data is a valuable good to be considered when assessing damages.^133^ The Spanish legal system, too, is reported to be leaning towards a pragmatic approach in deserving cases (rather than an established principle) on ownership of data. If the courts of these European countries were to affirm a property right, who owns it remains an open question. In the contexts of health information, courts might be strongly influenced by the common business understanding that the data owner is the entity or individual that generates the data, rather than the patient.^134^

Australia reportedly takes a position similar to the UK. Cases have generally found that trade secrets and know-how are not property, but the matter has not been settled.^135^ However, in a departure from the approach taken in the European Patent Convention, the majority of judges in the Australian High Court firmly refused to extend patent protection to isolated gDNA and cDNA sequences on the grounds that the essence of such claims lies not in the structure of those molecules but their naturally-occurring informational content, meaning there is nothing ‘artificially created’.^136^

New Zealand, on the other hand, has taken a more generous approach to property in information. In *Dixon v R*, the NZ Supreme Court expressed the view that digital files containing information could be regarded as personal property. It distinguished the digital file from both the information it contained and the medium upon which it was stored. In doing so, however, it was not clear the court meant to imply the existence of a separate property right in information.^137^

There are also differences between the USA and the UK but the distance is not wide. The primary difference is that US courts routinely suggest that trade secrets and confidential information are property and fully assignable.^138^ There is less doubt about this than one finds in the UK and other jurisdictions.^139^ Second, through legislation, one state (New Hampshire) deems information in a medical record to be the property of a patient,^140^ and five states (Alaska, Colorado, Florida, Georgia and Louisiana) deem genetic information to be the property of the individual from whom it came, although the statutory language has been criticized as ‘woefully imprecise’.^141^

Similar to the UK, US intellectual property laws treat copyrights and patents as property.^142^ Thus it is possible for hospitals and other businesses working with, or trading in data, to have property rights in data provided the information qualifies as intellectual property or a trade secret. Just like in the UK, patients in the US are unlikely to have such rights. And once made publicly available, information is unlikely to be considered confidential or secret, and loses its status as property. The US Supreme Court refused to grant patents over isolated gDNA sequences. Unlike the Australian High Court, however, it did not link this back to the informational nature of DNA. Its concern was to avoid pre-empting future innovation by effectively tying up a product of nature.^143^

As in the UK, US commentaries frequently state that information *per se* (ie, information that is neither a trade secret, confidential or covered by intellectual property rights) cannot be owned as property.^144^ Given its common law tradition, it is not inconceivable courts may provide a property right in information, possibly through some equitable doctrine,^145^ just as the court did in *Boardman v Phipps*.^146^ While the prospects are not promising, health information could, at the very least, acquire property-like status by feeding it through some cause of action that traditionally sounded in equity or by contracting into a property-type arrangement. Mixed signals from other courts also leave open the possibility that patients in some jurisdictions may have property-like interests in health and genetic information.^147^ But, in general, courts have been somewhat skeptical of providing property protection for such information.

The recent federal district court decision of in *Dinerstein v Google* illustrates this.^148^ In *Dinerstein*, patients of the University of Chicago Medical Center sued the University for sharing de-identified patient records with Google as part of a machine-learning research partnership, claiming, among the litany of causes of action, breach of contract, theft, and invasion of privacy. Three issues bear mentioning. First, the court rejected the plaintiff’s theory that appropriation of his electronic health records (‘EHRs’) created a cognizable harm for standing purposes.^149^ The reason: the plaintiff could point to no statute that created a property interest in his EHRs, and he made no real argument about why the common law would do so. Second, in dismissing the plaintiff’s claims for, among other things, breach of contract and unjust enrichment, the court noted that the plaintiff failed to show that he suffered any harm from the use of his personal health information without his consent—the information itself did not have ‘value’ the law was prepared to recognize. Finally, while the court declined to allow a claim for breach of confidentiality for unauthorized disclosure of a patient’s medical information, it noted that ‘a number of state courts have recognized such a tort’.^150^ However, the decision did not settle the question whether medical information *per se* can be treated as property. Indeed, the opinion did not discuss the property issue in any depth, in large part because it did not form a substantial part of the claimants’ case. The court ruled only that patients did not have a clear property interest in their data, not that a property interest in health information is inconceivable.

*Dinerstein* may hint at a general judicial skepticism towards medical information as property *per se*, but does not settle the issue in the US. In our view, however, it seems exceedingly unlikely that courts will fashion health information into property without the aid of some existing legal doctrine, probably an equitable one. This would place the limits of health ‘property’ alongside UK cases like *Boardman*, discussed above. That is, indeed, what some courts have begun to do by recognizing a cause of action for breach of confidentiality when there is an unauthorized disclosure of patient health information.

But that is not to say that US law lacks a possible method for recognizing property in health information. There is some caselaw that could facilitate this process as Professor Contreras explains in his article, *The False Promise of Health Data Ownership*.^151^ He cites a Ninth Circuit case, *Kremen v Cohen*, which developed a three-part test to determine whether an intangible (such as a license to operate a taxi) could properly be recognized as property:

First, there must be an interest capable of precise definition; second, it must be capable of exclusive possession or control; and third, the putative owner must have established a legitimate claim to exclusivity.^152^

The test is remarkably similar to that proposed in the English case *National Provincial Bank Ltd v Ainsworth,* or by the ones proposed Professors Aplin and Bently. While it omits the issues of notice, clarity on first entitlement, and relative stability, it usefully affirms the importance of the subject matter being precisely defined. And adds the point that the privileges of exclusivity should only be conferred for good reason. Without the latter point, property unjustifiably restricts other people’s liberty to access to non-rivalrous things—and many social harms follow. We address this point in Section V.

## IV. NON-PROPRIETARY PROTECTION OF PATIENT HEALTH INFORMATION

If patients are not considered owners of their health data, why does the law often require patient consent prior to the use of personal health data? And why, for example, are patients sometimes required to re-consent prior to medical research using data collected at an earlier time point? One reason is that the law protects health information through a variety of non-proprietary frameworks. In the UK, these include the law of confidentiality, the tort of misuse of private information (which is based on Art 8 of the Human Rights Act 1998), the EU General Data Protection Regulation (GDPR), contract law, the law of negligence, and the criminal law. While there is no independent law of informed consent in the UK, informed consent to the use or disclosure of health data is relevant in determining if another legal violation has occurred. This section demonstrates that a patient’s interests in informational privacy are protected in several ways, and that it is no accident that patients’ rights do not extend further in these legal frameworks. Accordingly, policy reform to recognize property in health information *per se* might (i) be redundant and add to legal complexity and fragmentation; or (ii) might significantly extend private rights of control, in which case a persuasive reason for doing should be identified. These points are the focus of Section V.

### IV.A. Medical confidentiality

Under the law of confidentiality, healthcare providers (public and private) generally owe a duty to protect the confidence of their patients.^153^ Providers must not misuse or divulge information disclosed in confidence without permission.^154^ This duty, however, is not absolute. Various considerations, which bear on the ability of modern healthcare systems to function, may override it.^155^ Courts have tried craft these considerations carefully by balancing public and private interests. Providers may disclose information when the law obliges disclosure (eg, to notify public health authorities, or to respond to a subpoena); when it is anonymized;^156^ when the patient expressly or implicitly consents; when it is in the public interest;^157^ or when it is necessary for the proper working of the hospital.^158^ Disclosure may be required, for example, to allow computer technicians to run IT systems and auditors to review financial reporting. There is also an avenue to set aside the common law duty of confidentiality under section 251 of the NHS Act 2006,^159^ which permits applications to be made to the Confidentiality Advisory Group (CAG) to share anonymized patient data with the Clinical Practice Research Datalink Service (CPRD), which is a department within the UK’s medical agency, MHRA. Guidance from the General Medical Council (GMC) offers further examples of permitted disclosures of confidential information in the medical setting (eg, when disclosure is needed for direct care or to protect others who may be at-risk).^160^

### IV.B. The tort of misuse of private information

Courts have recently developed a new area of law that protects informational privacy to ensure UK case law accords with Article 8 of the Human Rights Act 1998: the tort of misuse of private information. The law applies in circumstances where an individual has a reasonable expectation of privacy even if the information may not be confidential (ie, secret). If a medical professional reveals private medical information without permission, this may constitute both a breach of confidentiality and a misuse of private information.^161^ Patient consent is therefore central to the question of whether disclosure amounts to misuse of private information.

Private information is not considered to be misused if its disclosure was for a legitimate purpose. Article 8 of the Human Rights Act 1998 permits disclosure of private information where it is ‘necessary in a democratic society in the interests of national security, public safety or the economic well-being of the country, for the prevention of disorder or crime, for the protection of health or morals, or for the protection of the rights and freedoms of others’.^162^ Despite the broad language, in practical settings the boundaries of the public interest exception are vague and uncertain.

### IV.C. The General Data Protection Regulation (GDPR) and the Data Protection Act 2018

The EU GDPR, implemented into UK law by the Data Protection Act 2018, now represents one of the primary forms of protection of patient health information. It covers the processing of personal data, whether the data are in electronic or computerized records, or on paper. ‘[P]ersonal data means any information relating to an identified or identifiable natural person (“data subject”)’.^163^ Since the ways in which the data may become identifiable are broad (eg, by including names, identification numbers, location data, online identifiers, or one of several special characteristics in a data set), the GDPR applies in practice to information which is or can be assigned to a particular individual person in any way.

Health^164^ and genetic information^165^ are classified as ‘special categories’^166^ of personal data (along with data about ancestral origin, religious beliefs, political views, and sex life). Since special categories of data are considered particularly sensitive, the GDPR prohibits the use of these data except in specifically enumerated situations.^167^ Three such grounds for lawful processing of health information include where the data subject gives explicit consent;^168^ where processing in necessary for the provision of medical treatment or preventative strategies; ^169^ or where the data subject has made the data at issue public.^170^ Additionally, there are public interest grounds on which academic and hospital researchers may be able to justify the processing of health data without explicit patient consent. For example, where the processing is ‘necessary for scientific…research…or statistical purposes’,^171^ or where it ‘is necessary for reasons of public interest in the area of public health, such as…ensuring high standards of quality and safety of health care’.^172^

Regardless of whether consent is obtained or required, the GDPR contains a handful of cardinal principles that data controllers must observe when processing personal data. These include, amongst other things, the principles of accuracy, transparency, integrity, confidentiality, and security. Notably, these cognate principles protect interests related to, but different from, an individual’s informational privacy and autonomy. Personal data, the GDPR mandates, shall be accurate and, where necessary, kept up to date.^173^ To meet the transparency principle, summary information about the data controller, processors, data sharing and the purposes of processing must be provided as a matter of course and a copy of the personal data if the subject requests.^174^ The principles of ‘integrity and confidentiality’ are elements of data security. These require data controllers to take appropriate technical and organizational measures against unauthorized or unlawful processing of personal data and accidental loss, destruction, or damage.^175^ The principle of data security also requires appropriate steps are taken to pseudonymize and encrypt personal data, to store and process it in resilient ways, and to evaluate compliance with these systems.^176^ Fully anonymized data are not considered personal data. However, data will not be effectively anonymized if the organization in possession of the data could at any point use any reasonably available means to re-identify the individuals to which the data refers.

The legal rights that patients have under the GDPR are relatively strong. Individuals have a private right of action to enforce the GDPR or to claim compensation for damage or distress.^177^ (They lack such right under the US Health Insurance Portability and Accountability Act.^178^) The GDPR also envisages collective redress.^179^ Individuals can also complain to the Information Commissioner’s Office, which has powers to investigate and to issue orders and penalties. Violating the GDPR can result in serious consequences including the possibility of paying compensation, hefty penalties, and even criminal liability.^180^

### IV.D. Contract law

It is possible to protect patient health information using contract law. But the nature of this information poses a number of significant difficulties in the contractual context. First, transaction costs would be prohibitively high. Patients would need to contract with every person who used their data, and to do so in a way that placed limits on how their data could be used. Second, patients in the UK do not have contracts with the NHS, although they may have contracts with private providers.^181^ Third, there are significant power and informational asymmetries: patients are in a weak bargaining position relative to medical providers. If they want care from a provider, they are unlikely to spend time negotiating how the provider will use information about their health. They are also unlikely to have an experienced sense of what is likely to be a mutually acceptable amendment. And they are unlikely to know the details of future uses.^182^ This negotiation process also risks tainting a healthy provider-patient relationship, converting it to one of care to one of commercial bargaining over downstream uses of information about the patient. Finally, even where a contract does exist between a patient and a healthcare provider, it is often difficult to establish that the patient has suffered any compensable damage from the use of their data.^183^

### IV.E. The law of negligence

Keeping a patient’s affairs private is part of the duty of reasonable care owed by hospitals or doctors under the laws governing professional negligence. Thus a patient could sue a doctor or hospital that failed to take reasonable steps to keep sensitive health information secure. The patient may be disappointed, however, with the compensation awarded. The law of negligence rarely compensates pure psychiatric or psychological harm, or pure economic loss.^184^ Pain and suffering, and lost earnings are usually compensated only alongside physical harm.^185^ Generally speaking, a patient who merely felt embarrassed or violated by the unauthorized, negligent use of health information would not be eligible for compensation.^186^

In some circumstances, patients have sued a healthcare provider in negligence for failing to disclose a patient’s health information. In a recent case, a woman sued a hospital arguing that the hospital owed her, a patient’s daughter, a duty to warn her of a potential genetic disorder which she might have inherited from her father, the patient. She claimed that she would have terminated her pregnancy if physician had disclosed it to her, and that the hospital’s failure to tell her about her health risk was unreasonable.^187^ The English Court of Appeal held that doctors may be liable for not disclosing patient health information, at least in some narrow circumstances.^188^ This is an unusual feature of tort liability that does not arise in the law of confidentiality or the GDPR.

### IV.F. Criminal law

The criminal law of theft rarely applies to health information, largely because that information is not considered property that is capable of being stolen within the meaning of the Theft Act 1968.^189^ The Computer Misuse Act 1990, however, criminalizes ‘hacking’ into a database to access confidential information. As Herring explains, staff who have permission to access one part of a database may be guilty of this offence if they access another part that they are not authorized to access.^190^ Accordingly a healthcare professional is guilty of an offence is she accesses the hospital database to snoop on someone who is not her patient.^191^

### IV.G. The relevance and limits of informed consent

While informed consent is not a freestanding cause of action, consent is relevant to liability under the laws discussed above. In an action for breach of confidentiality, for instance, there is no misuse of confidential information if a patient consents to the way the health information is shared. In an action for negligence, the healthcare professional can argue that they acted reasonably (and therefore not negligently) if they use health information in accordance with the patient’s consent.^192^ In contract law, there is no breach of contract if the patient agreed to a particular use of information. Under the GDPR, patient consent is considered a legitimate basis for processing personal data and no other basis is thus required.^193^

Consent, if not always informed consent, therefore represents one of the main non-proprietary mechanisms by which patient interests in medical data are protected. As we have seen, however a delicate compromise is required to balance patient interests in medical data against wider public and third-party interests. In part, this is achieved by the fact that there is no single definition of informed consent. The meaning changes to reflect the scope of the duties, rights, and exceptions. The GDPR typically requires explicit, specific and informed consent.^194^ But there are many grounds for processing personal data without consent. Some of these reflect public interests, such as medical research, public health medicine and management of social care systems;^195^ and some reflect private interests, such as processing to support the legitimate interests of employers, litigants, and not-for-profit bodies.^196^ In the law of confidentiality, both express and implied consent, as well as broad consent,^197^ are legally valid forms of consent, and a defense applies where the use is necessary and proportionate to protect the public interest. Negligence law adjusts the material risks that must be disclosed prior to obtaining consent according to the patient’s circumstances, and very few exceptions apply once a duty to inform is established.^198^ All three areas of law are united by a common conceptual exception: it may be legal to disclose or use information even if the patient does not consent, including in healthcare settings, in order to balance patient interests in medical data against wider public and third-party interests.

## V. SHOULD PATIENT INFORMATION *PER SE* BE PROTECTED AS PROPERTY?

As described in Section III.A, there is no firm rule of law on whether patient information *per se* can be property. In current UK law, property rights in health information arise in the context of intellectual property law. Even here, though, property rights are unlikely to belong to the patient. Section IV demonstrated that other non-proprietary avenues provide patients with legal protection for their data, where consent (and refusal) is relevant but neither absolutely necessary nor authoritative. Critics argue that these options are inadequate, complicated, and overly fragmented.^199^ This leaves two questions open. Should the law recognize (i) property rights in health information *per se* (without the criteria of patents, copyright, or database rights), and, if so, (ii) patients as the owners of such property rights?

Authors have approached these questions in different ways. Some debate, on philosophical grounds, whether health information should be property (eg, labor theory, innovation theory).^200^ Others take a more practical approach and argue that ownership is inevitable (in various forms), and focus immediately on the question of who should own it.^201^ And some focus on particular shortcomings in health data regulation which they think patient ownership could mitigate. This section of the article offers a comprehensive synthesis of this subset of literature, and critiques the authors’ conclusions.

Scholars and policymakers claim five main advantages for recognizing property in health information *per se*. First, property in health information would better protect patients’ interests in informational self-determination (ie, autonomy and privacy). Second, propertization would increase market efficiency. Third, health information ownership by patients would give them a more equitable share of the financial benefits that flow from the growing profitability of the health data economy. Fourth, property in patient health information would clarify when and how information may be used by all actors in the medical system. Fifth, providing property rights in health information would encourage greater investment in health data-driven innovation. Through our analysis, we conclude, like other scholars have in similar contexts,^202^ that property, by itself, does not guarantee the achievement of any of these policy goals. We consider whether GDPR-style regulation of health information achieves the same goals just as well as, or better than, a proprietary-based system. Furthermore, we conclude that there are good reasons to think that property may in fact hinder, rather than promote, these policy aims.

### V.A. Health information ownership: to protect patients’ interests in self-determination?

Some argue that recognizing patient health data ownership would better protect patients’ rights to self-determination. Here property is a tool to safeguard patient privacy or autonomy (although the two are not always coextensive). The thought is this: patients who ‘own’ their health information will be better positioned to decide whether and under what circumstances information about them is used, accessed, sold, shared, etc.^203^ Their ‘consent’ will be essential. For example, Professor Laurie argues that rather than a right of ‘non-interference’ as provided by privacy, a property right would provide positive entitlement to ‘continuing control’, ^204^ and remedy the lack of privacy and data protection for anonymized information.

These arguments have several shortcomings. The first is that they are based on an over-simplified conception of property. As we have seen, the law cabins property rights in a variety of ways in order to account for competing interests. This becomes particularly acute in the healthcare context, where we have shown that patient data has a number of unusual features which demand special consideration. Property rights in patient data would likely be subject to significant limitations in order to take account of these features. The Human Rights Act 1998, for example, enables any property right in English law to be curtailed for the ‘general interest’.^205^

Second, contrary to Professor Laurie’s hopes, for logistical reasons if not for matters of principle, it is unlikely that patients who were the source of the anonymized health data could exercise property rights in it. Once anonymized, an individual simply cannot exercise control or self-determination; no one would know who to ask. And the individual may struggle to prove that the data emanated from them to enforce their rights. As a matter of principle, property rights in anonymized data would arguably exceed reasonable assertions. Professor Glenn Cohen poses an example where a physician draws on their cumulative experience from treating patients to write an account of a disease, or policy guidance. He argues it would be preposterous to require the physician to contact every patient they have seen with the condition, and ask their permission to use the composite knowledge generated by physician during the medical encounters.^206^

A third issue is that property rights in patient data might in practice resemble liability rules (rules which enable buyers to unilaterally remove entitlements provided they pay the value of the entitlement) because the third-parties can violate the property owner’s rights by paying a fee.^207^ This means that if the NHS violated a patient’s property rights in her health information by disclosing it without authorization but for a valid public interest purpose, the NHS could simply compensate her for the violation.

From this perspective, property rights for patients seem less attractive for those looking to safeguard autonomy and privacy. A patient’s property right in health information would be, like all other property rights, limited in scope.^208^ And these limitations seem unlikely to provide greater protection to a patient’s autonomy or privacy than that already provided by the GDPR, law of confidentiality, and Article 8 of the Human Rights Act. As shown above in Section IV, these frameworks accommodate tension between public and private interests, and do not give individuals absolute rights of control.

The relative change is perceived differently in the USA, where there is no full equivalent to the GDPR.^209^ Indeed, much of the literature arguing for property in data comes from the US. And, from this standpoint, it is probably best understood as an attempt to overcome the gaps in US data protection for personal information and health information.^210^ The Privacy Rule in the Health Insurance Privacy and Protection Act (‘HIPAA’)^211^ offers a framework of patient entitlements and protections similar to the GDPR^212^ and similar to what patients would enjoy if they owned their data.^213^ However it is missing both a right of private action for individual data subjects and widespread application to all data processers rather than to only ‘covered entities’.^214^ It may also benefit from a stricter definition of consent and, perhaps, stricter controls on permissible unauthorized disclosures.^215^ With these significant issues addressed, HIPAA’s privacy rule would provide broader protection than a property right, though it still may require further reform to address all of the interests health information governance should accommodate.^216^

A fourth problem with the idea that propertization of health information would strengthen a patient’s autonomous control is the issue of alienability. As discussed in I.B, a typical characteristic of property is that it is transferable. If health information is propertized, owners should be able to transfer it. A patient, for example, could sell or transfer their health information (ie, property) to other parties by an assignment or a license. And they might be inclined to do so if firms offered them money. Once assigned, the second party acquires the patient’s powers to deal with the property. Patients, however, occupy a poor bargaining position both in terms of power and knowledge.^217^ Patients are unlikely to foresee every and all possible uses to which their information may be put. And companies are likely to bargain for as much and as many uses as possible. Seen in this light, property rights could disempower patients with regard to downstream uses of their health information.^218^ In this case, recognizing property in patient health information likely would not improve patients’ rights to self-determination.^219^ As Professor Laurie has argued in the context of consent, property would give individuals ‘the illusion of power and control’ while the reality is far more bleak.^220^

One scholar, Professor Mark Hall, has proposed to remedy this problem by creating residual rights of control. These would be ‘default’ rules that protect patient interests from being overwhelmed by strong market players^221^ and require ‘opt-in’ to override.^222^ This requires, among other things, making some rights ‘nonwaivable or inalienable’—and ensuring that these features follow the information wherever it goes.^223^ This may remedy the problem of property being designed to be alienable, but leaves the purpose of recognizing property rights unclear. It would also require a significant degree of regulation and oversight to verify its correct operation—it would, in other words, be more like GDPR-style regulatory scheme. Perhaps, as Professor Julie Cohen argues, we should instead look to bolster privacy-enhancing norms through a new schema and set of laws centered around autonomy and privacy—something current property frameworks simply cannot provide.^224^

### V.B. Health information ownership: to support health data transactions and markets?

A typical justification for property rights in general is that they increase efficiency.^225^ Some commentators apply the same argument to the health information market.^226^ Their writing depicts a log-jam of interests in a stagnating health sector, with medical information currently lying ‘stunted in an undernourished field’^227^ waiting for property’s ordering and organizing force to be unleashed. The argument is not necessarily that *patients* should be recognized as the owner of the data, but that *someone* should be. Property ownership, on this view, will increase efficiency by creating investment incentives and clarifying obligations and duties relative health information. Incentives are arguably necessary for information because it is easy for competitors to copy and undercut the developer’s price.^228^ As mentioned in Section II.B, the idea is that property is advantageous because it entails rights *in rem* (ie, rights that ‘travel’ with the thing enforceable against any party) rather than mere rights *in personam* (ie, rights that travel with a particular person enforceable against parties to a contract).^229^

There is some force in this argument. Some property rights, particularly those that are evidenced by a registry (eg, the land titles registry or the patents register), create neat bundles of legal entitlements that can be signaled to others and used to facilitate transactions within the market. However, there are also significant problems with this line of reasoning. One is that introducing property does not always increase efficiency. For property to produce efficiency gains, it must have clear rules that demarcate the ‘units’ that can be transacted. In other words, what health information is protected, and who owned what parts of it, would need to be explained clearly.^230^ Although, as discussed in Section III.B, intellectual property laws can accommodate health information in their specialist forms of property, these laws include sub-rules and principles about ‘subject matter’, entitlement, criteria for protection, etc. In this way, intangibles such as expressions, inventions, databases, and trade identifiers can then be transacted. If we cannot increase efficiency by applying intellectual property concepts wholesale to health information, we would need to devise similar rules before making a judgment about property’s effect on social welfare. The heart of this issue is that for property in health information to be an efficiency generator it needs to be ‘exigible’.^231^ As discussed above in Section I.C, health information *per se* lacks features of exigibility.

Even assuming exigibility, highly diverse interests in information also cast doubt on the efficiency of exclusivity. A patient has interests in health information for seeking the right healthcare and the right time, for the right cost, and for the right level of protection of her protecting privacy, financial rights, and autonomy. The health provider has interests in treating the patient professionally, as well as promoting research, health care improvements and service efficiency. Commercial companies may seek to innovate competitively or simply to extract rents without regard to social welfare gains. Commercial data platforms have interests in acquiring and transferring information as service provision. It may be that none of these stakeholders actually requires exclusivity.

In some cases, exclusivity could hamper achieving these goals even more than it helps; a well-known disadvantage of proprietary exclusivity which has come to be known as ‘the tragedy of the anti-commons’.^232^ Each owner of exclusivity forecloses the other from the use of health information. The resulting fragmentation increases transaction costs, with extensive cross-licensing and license-stacking required whenever accumulations of information are needed. Since every seller can set her own price, the cost of successfully bargaining for each and every individual’s health information is relatively high. Thus, the full cost for transferring such rights may be prohibitive.^233^ Although bulk purchasing and other mechanisms could combat this problem, property does not clearly increase efficiency; and it could just as easily lead to an unending series of ‘toll booths’.

Some have responded to concerns about the tragedy of the anti-commons by advocating public ownership of health information.^234^ Professor Montgomery, for instance, argues that public ownership, through the NHS, would legitimize the protection and preservation of health information^235^ and enable productive uses without obtaining the consent or permission of each patient- or firm-owner, avoiding the net social loss created by fragmented ownership. Professor Rodwin makes a similar argument.^236^ Professor Hall adds the further argument that treating health information as publicly owned also enables network effects:^237^ the more individuals participate and use the information, the more valuable it becomes.^238^

One problem with the public ownership approach is that it requires the state to be a trusted mega-health-information owner—a proposition that makes some squeamish. In England, for instance, the outcry over care.data was substantial.^239^ The initiative merely provided the State powers to use data in certain ways, not powers to own data. The idea of the NHS owning health data is likely to be even more contentious. Illustrating this reluctance, considerable backlash occurred in 2017 when the NHS shared data with Google’s DeepMind,^240^ and again when deals were revealed between the Department of Health and large corporations in the US.^241^

Even assuming a trusted liberal democratic polity, public ownership would not be a panacea. One institution—even one with purest motivations and bureaucratic efficiencies—could probably not anticipate the appropriate value (or values) of information, or purchase the rights from thousands of property owners. Monolithic infrastructure is often too inflexible, or too bureaucratized, to respond to the changing needs of data users, processors, and researchers.^242^ In short, a single public owner would fall into the (many) problems affecting monopoly ownership. A single state owner would also sit awkwardly with one leg of its chair in the private sector. Beyond these problems, a single state owner will not solve market failures or capitalize on any network effects of a centralized database without a well-resourced technical infrastructure and the collection of useful information. The data it collects may be the most basic or the easiest to record, rather than the most useful. Particularly with the recent cuts to NHS, the state, at least in England, is unlikely to be well-positioned to meet the technical needs required to generate network effects.

Yet further problems confront the argument that informational property would increase efficiency by lubricating the gears of the health data market. Propertization—either for patients or firms—also carries risks to open communication and free speech.^243^ One risk is that propertization of health information may create property rights in ‘facts’ that intellectual property law is careful to avoid.^244^ Tying up facts would impede, rather than facilitate the pursuit of science, knowledge, medicine and free expression.^245^

Some believe ‘data trusts’ offer a better solution. Over the past 2 years, this idea has inspired a flurry of literature,^246^ public reports,^247^ consulting business, software developments, investment and pilot projects.^248^ The idea builds upon the legal tradition available in some countries for a legal ‘trust’ to hold property and for a trustee to manage the proprietary asset for a particular purpose. In its data incarnation, the ‘data trust’ and trustee would manage data or digital assets. And in the health sector, for example, its purpose would include maintenance of a registry of clinical data authorized for delegated management of secondary uses; long-term data governance that protects research subjects; complex value allocations among members of a data collaboration; and the isolation of data from a researcher’s insolvency risks.^249^ Professor Delacroix and Lawrence,^250^ and many others, argue that ‘data trusts’ also have advantages beyond more efficient data chain transactions. For example, they would better empower individuals through pooling data (ie, strength in numbers) and by providing expert management assistance.

Synthesizing the literature, it is important to realize that although data trusts have potential to provide such advantages, they are far from a ‘Panglossian’ solution for efficiency.^251^ Moreover, most important to this article, only a small minority of authors proposing ‘data trusts’ also propose that data, or rights in data, should be recognized as property. They are not in fact a property-based framework, and in this respect some authors consider them significantly different from traditional legal trusts.^252^ Typically data trusts are envisaged as a contractual or corporate framework.^253^ Accordingly, they are more aptly described as ‘trusted data management schemes’, rather than data trusts.^254^ A recent policy proposal from the European Commission refers to them as trusted ‘data intermediaries’, and proposes a default rule (with exceptions) which would prohibit exclusive rights to re-use data held by public-sector bodies.^255^

### V.C. Health information ownership: to improve benefit-sharing for patients in the data-economy?

A second argument offered for patient ownership of health information is its potential to distribute more equitably the financial benefits that flow from the health data economy. With the global value of health data predicted to reach $34.27 billion by 2022,^256^ this is potentially a high value proposition. In this vein, Purtova sees patient data ownership as the key to warding off hungry corporate behemoths.^257^ She worries that without patient ownership of health information, private power will appropriate, assert, and maintain ownership of it. Her argument is, in short: if patients do not own it, private power will.

She further argues that the initial legal entitlement will heavily influence how the property is eventually exploited. Without clarity as to the original owner, many will assert ownership,^258^ and the large and powerful players will be the most likely to succeed. Accordingly, she argues it is critical to allocate the entitlement clearly to the individual. Otherwise, individuals will be reduced to ‘data-meat’, slaughtered and traded on the open market. It is an argument based in power politics and market forces.

Other commentators argue similarly that property rights will give patients a better foothold in the data economy.^259^ They will be able to insist that some of the value associated with the information is passed to them; otherwise they can refuse to place their health information in the information market.

Despite their vigor, these arguments do not provide a convincing reason to recognize property rights in patient information. As noted above, the concept of property offers no clear advantages over and above other legal frameworks in which consent is relevant, such as the GDPR.^260^ Property is also subject to the same shortcomings as consent-based mechanisms for benefit-sharing. Scholars have noted, quite accurately, that providing property rights to patients themselves will do nothing to overcome the market realities that consumers face with inferior bargaining positions and knowledge asymmetries. They will no more leverage their property rights than their consent rights.^261^

### V.D. Health information ownership: to increase legal certainty for clinicians and health service providers?

Another argument for recognizing property in health information is that it would clarify the permissible boundaries of use. This is one of the main arguments offered by scholars who think property rights should be recognized in human biospecimens. They argue that property rights in tissue, for those from whom the tissue comes, would clarify what can be done with human tissue. Property law they point out is a field of law with a long history. Therefore, they argue, it could supply existing default rules that would remove some of the ambiguity about tissue transfers.^262^ Analogously, healthcare professionals often worry about when and how they are permitted to disclose health information. Frequently, they assume that answering the ownership question (who owns the information?) will clearly answer the disclosure question (can we disclose the information?).

Unfortunately, the assumption that property rights bring about greater legal certainty is not correct. The question of ownership does not map onto the question of permitted disclosure and creates additional questions that are likely to frustrate medical practice. Given the way non-proprietary legal frameworks operate,^263^ clinicians and healthcare providers may be permitted (or required) to disclose health information regardless of who owns it. Ownership is not the relevant question because disclosure is governed by a separate set of rules. Four examples illustrate:

(1) Doctors, we noted in Section IV, owe a legal duty of confidentiality to their patients. This is not premised on the patient ‘owning’ the information. It is premised on the relationship of trust; in other words, the circumstances in which the patient confided in the doctor or healthcare professional.
(2) Doctors, we noted in Section IV, have a professional duty under negligence law to provide a reasonable standard of care to patients, and to an extent to act reasonably so as to do no harm as a result of their medical actions. The law of negligence then may oblige a doctor in certain circumstances to disclose information to authorities, relatives, or sexual partners of the patient. None of these duties is premised on the doctor ‘owning’ the information, but rather a duty to take reasonable care to protect and help others.
(3) Legislation sets out situations when doctors are permitted or required to disclose health information for public health reasons. Ownership here is beside the point.
(4) The GDPR, as Section IV described, sets out an extensive list of rules and principles that regulate, and attempt to harmonize, the processing of personal data across the European Union. Within this framework, several rules authorize the use of personal data, including personal health data, for certain purposes. Notably, the rules authorize the use of personal data in some circumstances without making any references to who owns the personal data. The purpose for processing the data is highly relevant. The person to whom the data relates is also relevant, but, notably, their consent is not an absolute requirement. Their consent becomes a strict legal requirement only under the GDPR framework if none of the other lawful grounds for processing apply.

Since ownership of health information has no bearing on disclosure in any of the four legal situations just mentioned, introducing new rights of health data property and ownership rules would not improve legal certainty for health providers. The laws just mentioned would still need to be addressed and many of those laws would take priority regardless of who owned the health information.

Secondly, ‘property’ and ‘ownership’ are, as we indicate above, likely to create more, rather than less, confusion about when disclosure is allowed. One reason is because a property regime of patient health information would also need to import similar ‘safety valves’ that are built into non-proprietary laws that protect patient health information.^264^ These rules balance individual rights with the rights of other people, organizations, and the public interest. Much of the legal uncertainty in the current law derives from uncertainty about the precise point of balance. Even if a patient owns health information, for instance, the (hypothetical) law of health information property would probably allow a doctor to lawfully share the health information with organizations for the purposes of protecting serious threats to public health. It would also likely require the doctor to minimize the effect of sharing on the patient’s property right.

In sum, any potential unease healthcare professionals feel about disclosing information, or when it is permissible to do so, would not be assuaged by giving patients ‘property rights’ in this information. If anything, it is likely to complicate an already delicate area for healthcare professionals. Lawyers, doctors, researchers, and policymakers will be more effective in their efforts if they spend their time revisiting, revising, and following guidance for existing legal frameworks, rather than trying to define and limit property law in health data.^265^

### V.E. Health information ownership: to encourage data-driven health innovation?

A different concern is the idea that health information might not be generated unless property rights are offered as an incentive. This is a traditional economic argument based on the idea that health information is a public good.^266^ A public good has two features.^267^ First, a public good can be ‘consumed’ without reducing the quantity of the good (non-rivalrous). A book can be read, for example, without reducing the ‘amount’ of the book. Second, public goods are difficult (or impossible) to exclude people from the good (non-excludable).^268^ If one publishes a book, it is difficult to prevent other people from copying the book and selling it. This creates a ‘free-rider’ problem: since all books can be copied, consumers ‘of the privately provided public good’ have ‘a strong inducement…to try to be free riders’.^269^ They have an incentive, in other words, to copy and read the books without actually paying. Since public goods are easy to use and difficult to protect, incentives to invest in them are weak. Law intervenes to provide the protection, thereby creating the conditions necessary for investment in the public good.^270^

The argument echoes that made in relation to patents for drug development: patent-based property rights give the inventor exclusivity in the marketplace so she has the opportunity to price the invention to recoup the drug’s R&D costs without her price being undercut by competitors who merely copy the drug; without patent exclusivities, drug development would be economically unfeasible.

The argument is not particularly strong in the context of health information property. Health information appears to be generated in substantial quantities regardless of whether it is accompanied by property rights and market exclusivity. Indeed, the NHS (and private insurance companies and intermediaries) generate information as a matter of routine business. Offering these players incentives is pointless because no incentive is required to create the information.^271^

This line of reasoning, however, does not apply to all health information; certain kinds of health information might not exist unless there are proper incentives to create it. If a patient enters a hospital A&E or ER, for example, the hospital is unlikely to collect and record the patient’s weight, perform an eye examination, or analyze her DNA. Yet, this may be precisely the kind of information we need. Where and why the information is collected determines what information is collected; in other words, context controls collection.

This insight prompts one to ask: what kind of information do we want to generate, and is it being generated? Healthcare professionals may collect information by mistake, by design, or by combination of the two. When designed to collect information, entities will collect only the information that is relevant for the immediate (or reasonably foreseeable) purpose to which the information is put (or could be put). This means that there are certain kinds of health information that will not be generated at all in many cases unless a provider has a reason to collect it. It is theoretically possibly, then, to correct the problem by providing proper incentives. If we can identify market failures in valuable medical information, a property regime might help solve them.

An area that is arguably facing insufficient investment is not information, but the infrastructure needed for that information to be useful–infrastructure like big data management and interoperability.^272^ Huge quantities of health data exist, but are Balkanized with fragmented sources, multiple gate keepers, different technical platforms, even within a single provider like the NHS.^273^ To address this, healthcare providers, biobanks, and international research consortia are working on initiatives involving, for instance, processing systems for integrating data, coding systems, standardization protocols and a variety of other tools.^274^ Without this infrastructure, the information that is collected is much less useful.^275^ This is particularly true in systems where health data are fragmented and controlled by various private parties, as is the case in the USA. It is also an issue for the UK, if it wishes to share and access data across jurisdictions. Granting property rights in information *per se*, however, does not create the incentive to do so. Data property encourages collection and exclusion of certain kinds of information; and does not necessarily encourage the development of interoperable data or infrastructure to support it. Some companies may increase infrastructure development if organizing and managing data became more profitable.^276^ Again, however, the system would lack incentives to create interoperable data and platforms. More likely we would see—as we have in cable, telephone, and internet—an increasing effort by large companies to create exclusionary platforms that become the only place to collect data.

## VI. CONCLUSION

This article began with the observation that calls for patients and health services to ‘own’ or wrest control of ‘their’ data are becoming more insistent. These calls are often articulated as a need for patient rights of ownership or property in health information.^277^ While many individuals, organizations, and entities assert ownership in health information, many UK lawyers take the view that information *per se* cannot be the subject of property—and, therefore, therefore nobody owns health data.

Our research shows that the English legal system uses intellectual property laws to recognize property in information in special circumstances. Here, the property right is directed not so much at the information *per se*, but at a qualifying concept involving investment or creativity such as written expression, an invention or a database—and the underlying purposes for providing rights in the specific information. Patients would rarely be considered owners of health information. Doctors, companies, and data scientists, on the other hand, would qualify in some circumstances. Analyzing protection of information within the intellectual property framework also shows that any successful system of property in health information *per se* requires rules on eligible information, qualifying criteria, scope and duration of property protection, fair notice for third parties, exceptions from liability and remedies.

Outside intellectual property law, English courts have been hesitant to recognize property in information. There is not, however, an established principle in English law that information *per se* cannot be the subject of property rights. Instead, courts have been somewhat equivocal and reluctant to declare definitively whether health data *per se* can be owned as property.

Notwithstanding the lack of property rights in health information, there are many non-proprietary ways via which the law controls health information; the tort of misuse of private information (which is based on Art 8 of the Human Rights Act 1998), the EU General Data Protection Regulation (GDPR), contract law, the law of negligence, the criminal law, and the law of confidentiality (although some consider confidential information to be a type of intellectual property). Consent is highly significant in each of these legal frameworks, but it is not an absolute requirement for the use and disclosure of health information.

This background highlighted that there is no established legal principle against property in patient data, no strict legal rights for patient control of data, and considerable work involved in constructing a system for ownership and property in health data. In light of this, we considered whether the law should take this path. To evaluate this question, we reviewed and synthesized the literature, distilling the arguments within it into five policy goals that property rights in health information might serve. After reviewing these goals, we concluded that property is a poor means to advance them. It would not enhance patient self-determination, increase market efficiency, provide patients a foothold in the data economy, clarify legal uses of information, nor encourage data-driven innovation.

In reaching this conclusion, however, we recognize that health data processing poses real challenges in an increasingly data-driven economy. And that these challenges should be addressed. We think that the best way to address them is through a regulatory, not a property, framework.^278^ A regulatory model that addresses the policy goals in Section V has four components. First, it should set limits on how and when data can be used, and cognate issues such as transparency, data accuracy, security, and enforceability. This needs to account for the variety of interests in health data and its limited exigibility. The GDPR 2018 is one such model. It provides a significantly more sophisticated framework than the earlier EU Data Protection Directive 95/46/EC, balancing control between the data subject, the data controller, and public interests such as public health, medical research, national security, and journalism. Given that compensation awards are likely to be small for most individuals, the right to participate in a class action could be helpful.^279^

Second, there should be additional guidance from information law agencies, medical advisory, and licensing bodies, in consultation with lawyers and health professionals, to describe the conditions under which patient information can be disclosed. This will help to address current legal uncertainty about permitted and restricted disclosures. Third, we suggest developing interoperable data and platforms would be a far better support for innovation based on health data than recognizing property in data. Finally, we suggest a greater focus, and more research into, health data transactions based on a service model, rather than a goods model. In this way, transactions would focus on data flows as actions, rather than data as discrete assets.

Meanwhile, we urge ‘interdisciplinary tolerance’ for debates about ‘ownership’ of health data, by which we mean an understanding that the current language of ownership and property is mostly metaphorical.^280^ The language appeals to the notions of control and priority over something valuable, rather than indicating precise legal obligations.^281^ This, we think, accords with our second suggestion: better interdisciplinary communication. For this reason, and others we have discussed, we recommend moving the conversation away from data ‘ownership’ and towards a discussion about protecting individuals whose health information is processed, legitimate data uses/users, and a healthy and well-functioning data economy. Property is not essential to any of these issues. It may even inhibit our ability to grapple with problems of privacy, autonomy, economics, and morality in a clear way.

None of this prevents us from discussing health data as something with value, or even from trying to determine how to monetize it where appropriate. However, our approach does require us to talk more about what kinds of value it is assigned, and to whom and how that value flows. It also opens up new questions about how to monetize transfers of health data via contract law, if they are to be monetized, between various entities including companies, biobanks, patients, research participants, and intermediary data platforms. These are issues for further research.

## Footnotes

1 In fields such as genetic medicine, pediatrics, and infertility treatment, patients often form support groups on Facebook and Twitter where medical details are posted.

2 David Reinsel, John Gantz and John Rydning, *The Digitization of the World from Edge to Core*, InternationalDataCorporationWhitePaper #US44413318, 2018, https://www.seagate.com/files/www-content/our-story/trends/files/idc-seagate-dataage-whitepaper.pdf (accessed Dec. 4, 2019), at 11.

3 WiseGuy Reports, *Global Big Data in Healthcare Market Development and Demand Research Report—Forecast to 2022* (2016), https://www.wiseguyreports.com/reports/795043-global-big-data-in-forecast-to-2022 (accessed Dec. 4, 2019).

4
Academy of Medical Sciences, NewTechnologiesthatUsePatientData: SummaryReport of aWorkshopHeld on 24 April 2018 (2018), https://acmedsci.ac.uk/file-download/77418765 (accessed Feb. 15, 2020).

5 Jeffrey Ritter & Anna Mayer, *Regulating Data as Property*, 16 Duke L. & Tech. Rev. 220 (2018) at 221–223, 226–227.

6 Julia Powles & Hal Hodson, *Google DeepMind and Healthcare in an Age of Algorithms*, 7 HealthTech. 351 (2017); On behalf of the DeepMind Health Team et al., *Letter in Response to Google DeepMind and Healthcare in an Age of Algorithms*, 8 HealthTech. 11 (2018); Julia Powles & Hal Hodson, *Response to DeepMind*, 8 HealthTech. 15 (2018).

7 Laura Donnelly, *Security Breach Fears Over 26 Million NHS Patients*, TheTelegraph, Mar. 17, 2017, https://www.telegraph.co.uk/news/2017/03/17/security-breach-fears-26-million-nhs-patients/ (accessed Dec. 4, 2019).

8 Cara McGoogan, *NHS Sexual Health Clinic Fined £180K for Patients’ HIV Status Leak*, TheTelegraph, May 9, 2016, https://www.telegraph.co.uk/technology/2016/05/09/nhs-sexual-health-clinic-fined-180k-for-patients-hiv-status-leak/ (accessed Feb. 19, 2020).

9 Matthew Field, *WannaCry Cyber Attack Cost the NHS £92m as 19,000 Appointments Cancelled*, TheTelegraph, 2018, https://www.telegraph.co.uk/technology/2018/10/11/wannacry-cyber-attack-cost-nhs-92m-19000-appointments-cancelled/. For a list of other UK health data breaches, see the website medConfidential: Keep our secrets, https://medconfidential.org/for-patients/major-health-data-breaches-and-scandals/ (accessed Dec. 4, 2019).

10 For examples of misuse of biological specimens cited in U.S. literature, see Jessica L. Roberts, *Progressive Genetic Ownership*, 93 NotreDame L. Rev. 1105, 1124–28 (2018).

11 Daisuke Wakabayashi, *Google and the University of Chicago Are Sued Over Data Sharing*, N. Y. Times, Mar. 26, 2020, https://www.nytimes.com/2019/06/26/technology/google-university-chicago-data-sharing-lawsuit.html (accessed Feb. 4, 2020).

12 Sarah Knapton, *How the NHS Got It so Wrong with care.data*, TheTelegraph, July 7, 2016, https://www.telegraph.co.uk/science/2016/07/07/how-the-nhs-got-it-so-wrong-with-caredata/ (accessed Jan. 30, 2020).

13 See *infra* Section I.B.

14 Brittany Kaiser, *Tell Facebook: Our Data Is Our Property #OwnYourData*, change.org, https://www.change.org/p/tell-facebook-our-data-is-our-property-ownyourdata (accessed Dec. 4, 2019). *Cf.* Martin Mirchev, Iskra Mircheva, and Albena Kerekovska, *The Academic Viewpoint on Patient Data Ownership in the Context of Big Data: Scoping Review*, 22 J. ofMed. InternetResearch 1, 2–3 (2020) (explaining that ‘patient information ownership in the context of big data is a relatively new problem and apparently not yet fully recognized by the medical academic community.’).

15 Matt Hancock, *My Vision for a More Tech-Driven NHS,*MattHancock (Sept. 6, 2018) https://www.matt-hancock.com/news/my-vision-more-tech-driven-nhs (accessed Dec. 4, 2019); Nick Versel, *Topol Calls Patient Data Ownership a Future Civil Right* (2016) https://medcitynews.com/2016/10/topol-patient-data-civil-right/ (accessed Jan. 29, 2020); *Yearworth v North Bristol NHS Trust* [2009] EWCA Civ 37. Nick Versel, *Topol Calls Patient Data Ownership a Future Civil Right* (2016) https://medcitynews.com/2016/10/topol-patient-data-civil-right/ (accessed Jan. 29, 2020); *Yearworth v North Bristol NHS Trust* [2009] EWCA Civ 37.

16 Jorge L. Contreras, John Rumbold, Barbara Pierscionek, *Patient Data Ownership*. JAMA 935 (2018); Lothar Determann, *No One Owns Data*, 70 Hastings L.J. 1 (2019) (arguing that there should be no property rights in data).

17 In this context, a clinical encounter means an individual’s interaction with a healthcare provider, hospital, or other medical professional in their medical capacity for the purposes of medical advice, treatment, or care.

18
*See*, *eg*, Sonja Marjanovic et al., *Understanding Value in Health Data Ecosystems: A Review of Current Evidence and Ways Forward* 7 RAND HealthQuar. 3 (2018) (describing health data to include any health-related information that is of relevance to decision-making in a health system and that can inform prevention, treatment, cure, health promotion, self-care and wider public health activities, and decisions taken by stakeholders).

19 Since patients sign contracts and licensing agreements with third parties when they purchase or use such technology, many of these questions of ownership and legal rights may be decided using existing contract law. Due to space restraints, this article does not analyze how these rights interact with patient rights.

20
*See eg*, John Rumbold and Barbara K. Peirscionek, *What Are Data? A Categorization of the Data Sensitivity Spectrum* 12 BigDataResearch. 49–59 (2018) (Information is data that has been processed into a meaningful form). *See eg*, Olaf Dammann, *Information, Evidence, and Knowledge: A Proposal for Health Informatics and Data Science*, 10 Online J PublicHealthInform. e224 (2019). Information is generated from data by applying syntactical rules, which generate meaning by organizing or interpreting data. Since syntactical rules can vary, the information ‘hidden’ in health data is context dependent. For example, genetic data collections demonstrate variations between people, but what that means in terms of (health- or other-) information about those people is often far from clear. However, this Article does not engage further with these complexities. We will simply refer to any information about the patient as either ‘information’ or ‘data’.

21 These ‘special’ features of health information are not necessarily unique to health information. They are features that affect whether health information has traits suitable and susceptible to a property framework. In our view, other information probably has similar features that affect whether it would fit neatly under a property framework—but those issues are beyond the scope of this article.

22 Angela Ballantyne, *How Should We Think About Clinical Data Ownership*, 46 J. Med. Ethics 1 (2020) (quoting Kate Fultz Hollis, *To Share or Not to Share: Ethical Acquisition and Use of Medical Data*, 2016 AMIA Jt Summits Transl Sci Proc. 420 (2016)).

23
*Id*. at 2.

24 Amy L. McGuire, Jessica Roberts, Sean Aas, and Barbara J. Evans, *Who Owns the Data in a Medical Information Commons?*, 47 J. L. Med. & Ethics 62, 32–65 2019; Joke I. de Witte and Henk ten Have, *Ownership of Genetic Material and Information*, 45 Soc. Sci. Med. 51 (1997).

25 Kaiser, *supra* note 14.

26
*Patient Data Ownership ‘a Civil Right that’s yet to be Granted’, Says Topol*, Br. J. HealthcareComput. (2016), https://www.bj-hc.co.uk/patient-data-ownership-civil-right-thats-yet-be-granted-says-topol (accessed July 16, 2019); Versel, *supra* note 15; Katie Dvorak, *Why Health Data Ownership Is a ‘Civil Right’*, FierceHealthcare (2015), https://www.fiercehealthcare.com/it/why-health-data-ownership-a-civil-right (accessed Jan. 29, 2020).

27 J.H. Davis, *President Weighs In on Data From Genes*, The New York Times, Feb. 25, 2016, https://www.nytimes.com/2016/02/26/us/politics/president-obama-weighs-in-on-data-from-genes.html (accessed Jan. 11, 2019).

28 See further Section V.C. Jack Hardinges et al., *Data Trusts: Lessons From Three Pilots* (2019); Christopher Reed, BPE Solicitors & Pinsent Masons, *Data Trusts: Legal and Governance Considerations* (2019).

29 Sylvie Delacroix & Neil D Lawrence, *Bottom-up Data Trusts: Disturbing the ‘One Size Fits All’ Approach to Data Governance*, Int. DataPriv. L. ipz014 (2019).

30 Daniela Gunz et al., *Healthbank—Your Global People-Owned Health Data Transaction Platform*, 13 Orphanet J. RareDis. 17 (2018).

31 Genomics England, *Intellectual Property Principles for 100,000 Genome* 2 (2016) intellectual property principle 2. The point is qualified in relation to genome sequences: ‘Genomics England does not seek to own any individual’s genome sequences. Where this policy refers to ownership or licensing of assets in the context of genome sequences, such references are intended as a reference to ownership or licensing of rights to data and other intellectual Property which subsists in, claims and/or covers genome sequences.’; Genomics England, *Genomics England Intellectual Property Policy* 7 (2017).

32 Biobank Sverige, *Agreement on the Transfer of Human Biological Materials* 2 (2019), 3 Transfer of Biological Materials and Personal Data; Generation Scotland, *Data/Material Transfer Agreement* 3 (2018), 1.2 Grant and Scope.

33 Million Women Study, *Million Women Study Data Transfer Agreement* 4 (2015), 9 Reservations; Australian Prostate Cancer BioResource, *Material Transfer Agreement* 3, 7.2 Property & Rights; Australian Breast Cancer Tissue Bank, *Material Transfer Agreement* 6 (2010), 4 Ownership Rights; China Kadoorie Biobank, *CKB Data Access Agreement* 7 (2015), 7 Intellectual Property; Latvian Biomedical Research and Study Centre, *Material and Data Transfer Agreement* 3, 3 Rights; International Agency for Research on Cancer, *Material Transfer Agreement* 3 (2014), 3 Rights; Karolinska Institutet, *Agreement on the Transfer of Human Biological Materials* 2 (2016), 3 Transfer of Biological Materials and Personal Data; Indian Sapien Biosciences, *Material Transfer and Research Collaboration Agreement* 6, 5 Ownership and Rights; THL Biobank, *General Terms of Access* 5 (2018) Ownership and Intellectual Property.

34
Daniel Glazer, HenryLeibowitz andJasonGreenberg, Data as IP andDataLicenseAgreements 12 (2017).

35 Roberts, *supra* note 10, at 1129. The updated version accessed from Europe/UK no longer includes this statement.

36 Ballantyne, *supra* note 22, at 1. As Ballantyne notes, an equally common refrain is: ‘It is hard to know…who really owns the data’.

37
*Id.*

38 See below Section V.A. For those who argue that ownership will increase control of data, see eg Nadezhda Purtova, *The Illusion of Personal Data as no One’s Property*, 7 L. Innov. Tech. 83–111 (2015); Jonathan Montgomery, *Data Sharing and the Idea of Ownership*, 23 TheNewBioethics 81–86 (2017); Marc A. Rodwin, *The Case for Public Ownership of Patient Data*, 302 JAMA 86 (2009); M.A. Hall, *Property, Privacy, and the Pursuit of Interconnected Electronic Medical Records*, 95 Iowa L. Rev. 631–663 (2011).

39 Annie Sorbie, Wifak Gueddana, Graeme Laurie, David Townend, *Examining the Power of the Social Imaginary through Competing Narratives of Data Ownership in Health Research*, 68 J. of L. & Bioscis. 1, 5–6 (2021).

40 Sometimes the relationship works the other way, ie, property is everything that is owned, and ownership means having the right to exclusive use, etc. Determann, *supra* note 16, at 6 (“Ownership’ generally refers to ‘[t]he right to exclusive use of an asset’ or ‘the full right to dispose of a thing at will.’ Ownership assigns a thing to a person or legal entity and signifies that the object belongs to that person. We also use the term ‘ownership’ more broadly in everyday language with respect to owning an ability or responsibility, where one can ‘own up to’ having done something.’ [quoting JohnBlack, A Dictionary ofEconomics (5th edn, Oxford University Press 2017).].

41 For summaries, see Ivan Stepanov, *Introducing a Property Right Over Data in the EU: The Data Producer’s Right—an Evaluation*, 34 Int’lRev., Comps. andTech., 65, 68–70 (2020); Tanya Aplin, *Confidential Information as Property?* 24 Kings L. J. 172 (2013); MG Bridge et al, TheLaw ofPersonalProperty (Sweet & Maxwell 2013); James E Penner, TheIdea ofProperty inLaw (Oxford University Press 2003). There is a very lively debate about whether property really is something unique or whether it reduces to some essential qualities. *For example,* Henry Smith, *Property as Law of Things*, 125 Harv. L. Rev. 1691 (2012).

42
*Id.* at 336, 341 (noting that ‘the inclusion of powers of alienation and free exchange along with exclusive use is perhaps characteristic of the modern Western conception of ownership’ and that other competing systems of property may exist).

43
*See*, *eg*, TanyaAplin et al., Gurry on breach of confidence: the protection of confidential information (2nd edn 2012) (noting that the ‘normal sense [of the word property]…confer[s] an exclusionary right which operates against the whole world.’) The attributes of property are fluid and have constantly been adapted through common law or explicit regulation; Julie Cohen, *What Kind of Property Is Intellectual Property?*, 52 Hous. L. Rev. 691 (2014).

44
*For example*, Frances Plimmer, *Compulsory Purchase and Compensation: An Overview of the System in England and Wales,* 3 Nordic J. Surveying & RealEstateResearch 144 (2008); KeithDavies, Law ofCompulsoryPurchase andCompensation (Butterworths 1984); U.S. Constitution, Amendment V, (‘[P]rivate property [shall not] be taken for public use, without just compensation.’); *Knick v Township of Scott* (2019) 139 SCt 2162.

45
*For example*, The Patents Act 1977 (as amended), s.60; U.S. Patents Act, 35 U.S.C. § 271 (2019).

46 Some, particularly those who take an economic perspective, do not think this feature central. Purtova *supra* note 38. Purtova, however, confuses the idea of a property *right* with the *concept* of property. The former exists in virtue of legal rules (natural or positive); the latter exists as a tool for analysing relations between people, objects, and things in economics.

47 Or simply to reduce transaction costs. Smith, *supra* note 41.

48 Václav Janeček, *Ownership of Personal Data in the Internet of Things*, 34 Comput. L. & Secur. Rev. 1039–1052 (2018).

49
Jeremy Waldron, What Is Private Property 316 (1985).

50 Néstor Duch-Brown, Bertin Martens and Frank Mueller-Langer, *The Economics of Ownership, Access and Trade in Digital Data*, EuropeanCommission, JRC TechnicalReports: JRC DigitalEconomyWorkingPaper 2017–01 (2017) at 12 https://ec.europa.eu/jrc/sites/jrcsh/files/jrc104756.pdf (accessed Dec. 6, 2019). This is also referred to as the ‘legal realist’ view of property.

51
*For example*, Max Planck Institute for Innovation and Competition, *Arguments Against Data Ownership: Ten Questions and Answers* (2017) 1 https://www.ip.mpg.de/fileadmin/ipmpg/content/forschung/Argumentarium-Dateneigentum_eng.pdf (accessed July 1, 2020). (‘It is a common misconception that an owner of a data generating device [eg, a mobile phone manufacturer or a car manufacturer] can “own” data in a legal sense…A “data ownership right” does not currently exist either at EU or Member State level, or any other industrialized country.’).

52 ‘Persons’ here refers to ‘legal persons’ and therefore includes business entities, governments, etc., as well as individuals.

53 This point regarding property theory has been made in greater detail elsewhere. *For example*, Jane B. Baron, *Property as Control: The Case of Information*, 18 Mich. Telecomm. & Tech. L. Rev. 367 (2012) (arguing that arguments about control over personal information reveal problems with applying property theory to resolve issues of control of digital information).

54 [1966] 2 AC 46.

55
*Id.* at 127–128.

56
*Id*.

57
*Id.* at 107 (Lord Hodson) [holding information could be property (eg know how) but that was immaterial in this case]; *Id.* at 103 (LJ Cohen) (holding that the information obtained was not property in a ‘*strict* sense’) (emphasis added).

58
*Id.* at 89–90. Although on the particular facts, without much explanation, Lord Dilhorne found the information at issue not to be property of the trust.

59
*Id.* at 115.

60
*Id.* at 107, 110–11.

61
*Boardman v Phipps* [1964] 1 WLR 993, at 1012.

62
*Id*. at 1012; Alastair 85, *Equity, Confidentiality and the Nature of Property* in Helena Howe and Jonathan Griffiths (eds), Concepts of Property in Intellectual Property Law (Cambridge University Press 2013) at 110–114.

63 Often the Lords use the words ‘property’ and ‘asset’ interchangeably. See *Boardman v Phipps*, *supra* note 54, at 93 (LJ Dilhorne); *Id.* at 111 (LJ Hodson).

64
*Boardman v Phipps*, *infra* note 71 at 63 (‘In the present case the information belonged to the trustees only if the appellants were their agents.’) (LJ Dilhorne).

65
*Id.* (quoting *Aas v Benham*, [1891] 2 Ch. 244, 255–256 C.A. [emphasis added]; For a similar view, Emily Hudson, Philips v Mulcaire [2012]: *A Property Paradox, in*Landmark Cases in Property Law n. 91 (Simon Douglas, Robin Hickey, & Emma Waring eds., 2015) [last sentence of section 2.2].

66
*Fairstar Heavy Transport NV v Adkins* [2013] EWCA Civ 886, at [49] (“Whether there could be property in information—whether the plaintiff had ‘a proprietary right in the content’—‘was not the … point.’).

67
*Id.* at [48].

68 [2014] EWCA Civ 281.

69
*Id.* at [15].

70
*Id.* at [27].

71
*Your Response Ltd v Datateam Business Media, supra* note 68 at [39]–[42]. For example, Lord Justice Davis was concerned that allowing a common law lien over databases could disrupt settled expectations within the IT industry. Lord Justice Floyd was concerned that recognizing information as subject matter of property could have a variety of unexpected consequences.

72
*Id.* at [42].

73
*Douglas v Hello* [2013] EWHC 786 at [275].

74 Cf Hudson, *supra* note 65. Lionel Bently suggests that Lord Upjohn’s view has been widely preferred because he was the more senior judge, and because it accords with other opinions from academics and judges. Lionel Bently, *Trade Secrets: ‘Intellectual Property’ But Not ‘Property’? in*Concepts ofProperty inIntellectualPropertyLaw 68, 80–81 (H. Howe and J. Griffiths eds., Cambridge University Press 2013).

75 Contreras shows that U.S. courts have also been willing to treat ‘intangibles’ as property in a variety of contexts. Jorge Contreras, *The False Promise of Health Data Ownership*, 94 N.Y.U. L. Rev. 624, 634–35 (2019) [hereinafter *Health Data Ownership*].

76
Lionel Bently et al., Intellectual property law 1–3 (2018).

77 Bently, *supra* note 74, at 70–76.

78
*Id.* at 80–81. Paradoxically then, confidential information is intellectual property, but not property. Tanya Aplin, Lionel Bently, Phillip Johnson, and Simon Malynicz, eds. Gurry, on Breach of Confidence: The Protection of Confidential information (2012).

79
Bently et al., *supra* note 76, at 3–4.

80
*Ladbroke (Football) v William Hill (Football)* [1964] 1 WLR 273, at 281, 289; *Independent Television Publications Ltd. v Time Out Ltd.*, FSR 64 (1984).

81
Bently et al., *supra* note 76, at 98–99.

82
*Eg*, *Feist Publications, Inc. v Rural Telephone Service Co*., 499 U.S. 340 (1991).

83
*See*, *eg*, *Fitbit Terms of Service*https://www.fitbit.com/uk/legal/terms-of-service (accessed Jan. 3, 2020): Fitbit’s Rights: “Fitbit Content” includes any photos, images, graphics, video, audio, data, text, music, exercise regimens, food logs, recipes, comments, software, works of authorship of any kind, and other information, content, or other materials that are posted, generated, provided, or otherwise made available through the Fitbit Service.’

84
*See eg*, *id.*: ‘Fitbit Content, the Fitbit Service, and its underlying technology are protected by copyright, trademark, patent, intellectual property, and other laws of the United States and foreign countries. We reserve all rights not expressly set out in these Terms.’

85 See eg Copyright, Designs and Patents Act 1988 s.3(2).

86
*Id.* s.3A. Directive 96/9/EC of the European Parliament and of the Council of Mar. 11, 1996 on the legal protection of databases. The UK enabling legislation is Copyright and Rights in Databases Regulations 1997/3032 (‘Database Regulations’).

87 Database Regulations s.3A.

88
*Id.* s.13 (‘substantial investment in obtaining, verifying, or presenting the contents of the database.’); Directive 96/9/EC of the European Parliament and of the Council (‘EU Data Directive’) Art. 1 s.2 (‘database’ shall mean a collection of independent works, data or other materials arranged in a systematic or methodical way and individually accessible by electronic or other means.’); EU Data Directive Art. 7 sec. 1 (‘right for the maker of a database which shows that there has been qualitatively and/or quantitatively a substantial investment in either the obtaining, verification or presentation of the contents to prevent extraction and/or re-utilization of the whole or of a substantial part, evaluated qualitatively and/or quantitatively, of the contents of that database.”).

89 Database Regulations s.13(1). Affirmed in *Health & Case Management Limited (HCML) v The Physiotherapy Network Limited* (TPN) [2018] EWHC 869 (QB), at [91]. Neither the legislation nor the case define ‘property right’.

90 DLA Piper, *Rights in Data Handbook* (2013) at 14, https://www.dlapiper.com/en/finland/insights/publications/2013/01/rights-in-data-handbook-2013 (accessed Dec. 6, 2019).

91 Database Regulations s.16; EU Data Directive Art. 7 s.1.

92 Database Regulations s. 20.

93 EU Database Directive para. 46.

94 DLA Piper, *supra* note 90, at 12.

95 Data Regulations ss.14(3)–(4) (defining the maker of a database to include ‘Her Majesty’ when the database is ‘made by Her Majesty or by an officer or servant of the Crown in the course of his duties’ and also in instances to the House of Commons and the House of Lords).

96
*See also* European Patent Convention (EPC) Art. 74, and Chap. IV.

97 Patents fundamentally protect *knowledge of* particular inventions. See Talha Syed, Physicalism of Patent Law (working paper 2020).

98
*Id.* Art 69 (extent of protection); Patent Act of 1977 (PA77) s.125 (extent of invention).

99 Any claim that embraces a product or process that fails any of these requirements can be declared invalid by a UK court at any point in the patent’s life. Other valid claims are unaffected, provided they are not dependent on the revoked claim.

100 PA 77 s.7(3).

101 European Patent Convention Article 54(4) and (5).

102 David A. Simon, *Off-Label Innovation*, 56 Ga. L. Rev. __ (forthcoming 2021).

103 Directive 98/44/EC on the Legal Protection of Biotechnological Inventions, Art 5(2).

104 PA 77 s.60(1)(a).

105
*Id.* ss.60(1)(b), (c).

106
*Actavis UK Ltd v Eli Lilly & Co* [2017] UKSC 48.

107
*Id.* s. 60(5)(b).

108 Bently, *supra* note 74, at 60; Aplin, *supra* note 41.

109 Trade Secrets (Enforcement, etc.) Regulations 2018 s. 3(1).

110 EU Trade Secrets Directive (2016/943). Although not mandatory under the EU Directive, in the UK the law of confidentiality and trade secrets does not limit the ability of those holding trade secrets or confidential information from disclosing, for reasons of public interest, information, including trade secrets, to public, administrative or judicial authorities for the performance of the duties of those authorities. *Lion Laboratories Ltd v Evans* [1985] Q.B. 526, [1984] 3 WLUK 239; *X Health Authority v Y* 1988] 2 All E.R. 648; *Campbell v Mirror Group Newspapers Ltd* [2004] UKHL 2;2 EU Trade Secrets Directive (2016/943), Art. 2(1)(a)-(c); Trade Secrets (Enforcement, etc.) Regulations 2018 s.2. The EU regulations specifically enumerate cases in which trade secrets may be disclosed: exercising freedom of expression and information; revealing wrongful or illegal activity to protect the public interest; exercising legitimate authority by representatives after disclosure by workers under Union or national law; and protecting legitimate interest recognized by the EU or national law. EU Trade Secret Directive Article 2(b).

111
Aplin et al., *supra* note 78, at 316–324.

112 [2010] EWCA Civ 1214.

113
Aplin et al., *supra* note 78, at 311–312, 8.03; Aplin, *supra* note 41; Cf Hudson, *supra* note 65.

114
Aplin et al., *supra* note 78, at 328. Aplin notes that this is true provided the obligation of confidence is ‘not personal in nature (ie, where the identity of the person to whom the obligation is owed is a matter of importance to the party on whom the obligation rests).’ *Id.* at 328–29.

115
*See id.* at 313 s.8.05.

116
*Coco v AN Clark (Engineers) Ltd* [1968] FSR 415 (quoting *Saltman Engineering Co. Ltd. v Campbell Engineering Co. Ltd.* [1948] 65 RPC. 203, at 215).

117
*Douglas v Hello*, *supra* note 73 at [184].

118
*See supra* Section III.B4 for more on medical confidentiality.

119
*See supra* Section III.A.

120
*Z v Finland* [1997] ECHR 25; *Campbell v MGN Ltd* [2004] UKHL 22 at [145]; *Australian Broadcasting Corporation v Lenah Game Meats Pry Ltd* [2001] 208 CLF 199 at [42] (judgment of Gleeson CJ)*.*

121
Michael Bridge, Louise Gullifer, Kelvin Low, Gerard McMeel, eds., The Law of Personal Property paras 1–064 (2021).

122 [1965] A.C. 1175, 1247–1248, HL (emphasis added). This starting point was also adopted in the case of *Armstrong DLW GmbH v Winnington Networks Ltd* [2012] EWHC 10 (Ch), [2013] Ch 156, [2012] Bus LR 1199, paras [42] and [50].

123
*See* Christopher Buccafusco & Mark A. Lemley, *Functionality Screens*, 103 Va. L. Rev. 1293 (2017).

124 Formal registration of copyright is an option in the U.S. It affords certain advantages in any litigation due to the notice provided by registration.

125 There are few defenses for infringement of a database right. But scientific research is one.

126 Contreras, *Health Data Ownership*, *supra* note 75, at 636–638 (explaining challenge of defining individual health information). *See also* Ellen Wright Clayton, Barbara J. Evans, James W. Hazel, and Mark A. Rothstein, *The Law of Genetic Privacy: Applications, Implications, Limitations*, 6 J. of L. & Bioscis. 1, 8 (2019) (noting the problems associated with defining *genetic* information).

127
*Id*.

128
Hugh Breakey, IntellectualLiberty: NaturalRights andIntellectualProperty at 75–96 (2015) (user’s rights and the public domain). *See* David A. Simon, *Reasonable Perception & Parody in Copyright Law* 2010 Utah L. Rev. 779 (2010); David A. Simon, *The Confusion Trap: Rethinking Parody in Trademark Law*. 33 Wash. L. Rev. 1021 (2013).

129 Contreras, *Health Data Ownership*, *supra* note 75, at 657.

130 Professor Contreras recommends that a system of property in health information not be established because of the negative effects for data-driven research. *Id.* at. 647, 654 and 656. We reach the same conclusion but via a different path. In Section V, we review the five strongest arguments in favor of property in health data and find them unpersuasive. We agree that the existing protections for health information provide better systems for protecting the interests patients have in their health information.

131 Stepanov, *supra* note 41, at 73–74.

132 European Commission, *Legal Study on Ownership and Access to Data* 45 (2016) (citing P. Berlioz, *Consécration du vol de Données informatiques. Peut-on Encore Douter de la Propriété de L’information ?* 4 Revue desContrats 951 (Dec. 1, 2015)).

133
*Id.* at 50–56.

134
*Id.* at 73.

135
Aplin et al., *supra* note 78, at 311 n. 4.

136
*D’Arcy v Myriad Genetics, Inc* [2015] HCA 35, 89.

137 Bridge et al., *supra* note 121, at para 9–045. The case has been criticized: K. F. K. Low & D. Llewelyn, *Digital Files as Property in the New Zealand Supreme Court: Innovation or Confusion?* 132 L. Quart. Rev. 394 (2016).

138
Aplin et al., *supra* note 78, at 336, citing *Board of Trade of the City of Chicago v Christie Grain & Stock Co.* (1905) 198 U.S. 236, 25 S Ct 637 and cases following this case. See also *id* at 311 (including citations at n 3), 339 (including citations at n. 174 and 175.

139 Aplin *et al* personally doubt that the Supreme Court decisions regularly cited in support of trade secrets and confidential being property did indeed stand for that proposition, but accept that it is the popular view in the U.S. *Id.* at. 339.

140 McGuire, Roberts, Aas, & Evans, *supra* note 24, at 65.

141 Contreras, *Health Data Ownership*, *supra* note 75, at 641. *See also id.* at 627 n. 8 (noting that a sixth state (Oregon), which was the first to enact legislation recognising a property interest in genetic information, repealed its legislation in 2001); Leslie E. Wolf et al., *The Web of Legal Protections for Participants in Genomic Research*, 29 HealthMatrix 1, 3 (2019). Roberts, *supra* note 10, at 1128 (noting that an additional four states introduced unsuccessful bills: South Dakota, Alabama, Massachusetts and Texas).

142 The U.S. lacks a database right outside of copyright: see generally 17 U.S.C. § 101 et. seq.

143 The US Supreme Court held, ‘[a] naturally occurring DNA segment is a product of nature and not patent eligible merely because it has been isolated, but cDNA is patent eligible because it is not naturally occurring’: *Association for Molecular Pathology v Myriad Genetics, Inc*. 569 U.S. 576 (2013). The Supreme Court showed similar concern to avoid pre-empting future innovation with patents on abstract ideas and natural laws: Mateo Aboy et al., ‘*How Does Emerging Patent Case Law in the US and Europe Affect Precision Medicine?’*, 37 Nature biotech.1118–1120 (2019).

144
*See, e.g.*, Contreras, *Health Data Ownership*, *supra* note 75, at 631–32.

145 For instance, in *INS*—the majority expressed substantial doubt that information making up the ‘news of the day’ could be the subject of private property. However, it suggested that it might be considered ‘common property’ of the public; and also held that the complainant news distributor had a quasi-property right against a rival news distributor which unfairly misappropriated news which it had collected and published.

146 See *supra* Section III.A.

147
*Eg*, Peerenboom v. Perlmutter, No. 2013-CA-015257 (Fla. Cir. Ct. Jan. 23, 2017); Cole v. Gene by Gene, Ltd., No. 1:14-CV-00004-SLG, 2019 WL 2571244, at *1 (D. Alaska June 21, 2019). See Roberts, *supra* note 10, at 1109–10 (discussing cases); Wolf et al, *supra* note 141, at 44; McGuire, Roberts, Aas, & Evans, *supra* note 24, at 65.

148 Dinerstein v. Google, LLC, 484 F. Supp. 3d 561 (N.D. Ill. 2020).

149
*Id.* at. 577–78.

150
*Id.* at. 594–95.

151 Contreras, *Health Data Ownership*, *supra* note 75, at 624, 635–36.

152
*Id.* at 636 (quoting Kremen v. Cohen, 337 F.3d 1024, 1030 (9th Cir. 2003) (citing G.S. Rasmussen & Assocs., Inc. v. Kalitta Flying Serv., Inc., 958 F.2d 896, 903 (9th Cir. 1992)).

153 Medical confidentiality is discussed in this section (non-proprietary protection) given that confidential information is arguably non-proprietary (see above).

154
Emily Jackson, MedicalLaw: Text, Cases, andMaterials 1, 420 (5thedn. 2019).

155
*Id.* at 421–422.

156
*R. v. Dep't of Health ex parte Source Informatics Ltd.*, 1 All E.R. 786, at [2].

157
*W v Edgell* [1990] 1 All ER 835 (threat by a mentally ill patient to seriously harm others); *Campbell v MGN Ltd*, *supra* note 120; See discussion of various public interest reasons in JonathanHerring, MedicalLaw andEthics (5th edn 2014) at 240–249.

158
*R. v Source Informatics Limited*, *supra* note 156 (Simon Brown LJ); see Jonathan Herring, Medical Law and Ethics (2006), at 240.

159 CAG is useful for collections of data but does not help in individual cases. E.g., ABC v St George's Healthcare NHS Trust [2020] EWHC 455 (QB).

160
General Medical Council, Confidentiality: GoodPractice inHandlingPatientInformation (2017), https://www.gmc-uk.org/static/documents/content/Confidentiality_good_practice_in_handling_patient_information_-_English_0417.pdf (accessed Dec. 5, 2019). Patient consent is often a key component in determining whether any particular disclosure is permissible. This does not always mean, however, that the patient affirmatively agrees to the disclosure—consent can be express or implied.

161
Herring, *supra* note 158, at 224.

162 Human Rights Act 1998.

163 The term is defined in Art. 4(1) of the GDPR.

164 GDPR Art. 4(15) (“[D]ata concerning health’ means personal data related to the physical or mental health of a natural person, including the provision of health care services, which reveal information about his or her health status.’). *See also* GDPR Recital 35.

165
*Id.* Art. 4 (13) (“genetic data’ means personal data relating to the inherited or acquired genetic characteristics of a natural person which give unique information about the physiology or the health of that natural person and which result, in particular, from an analysis of a biological sample from the natural person in question.’) See also GDPR Recital 34.

166
*Id.* Art. 9(1).

167
*Id.* Art. 9(2).

168
*Id.* Art. 9(2)(a).

169
*Id*. Art. 9(2)(d).

170
*Id.* Art. 9(2)(e).

171
*Id.* Art. 9(2)(j).

172
*Id.* Art. 9(2)(i). The precise scope of this provision has been criticized for lack of clarity: Miranda Mourby et al., *Governance of Academic Research Data Under the GDPR—Lessons from the UK*, 9 Int. DataPriv. L. 192–206 (2019).

173 GDPR Art. 5(1)(d).

174
*Id.* Arts. 12–15.

175
*Id.* Art. 5(1)(f).

176
*Id.* Art. 32.

177
*Id.* Art. 79, 82; Data Protection Act 2018 s. 167–169.

178
*For example*, Acara v. Banks, 470 F.3d 569, 571 (5th Cir. 2006) (holding that HIPAA does not create a private right of action); Jenna Becker, *Insufficient Protections for Health Data Privacy: Lessons from Dinerstein v Google*, Bill ofHealth (Sept. 28, 2020) https://blog.petrieflom.law.harvard.edu/2020/09/28/dinerstein-google-health-data-privacy/ (accessed Dec. 8, 2020); Joshua D.W. Collins, *Toothless HIPAA: Searching for a Private Right of Action to Remedy Privacy Rule Violations*, 60 Vand. L. Rev. 199 (2007). *See also* Dinerstein, 484 F. Supp. 3d at 575–76 (holding that analogous Illinois state statute did not create a private right of action).

179 GDPR Art 80; Data Protection Act 2018 (UK) ss. 187 and 188.

180 Data Protection Act 2018 (UK) Part 6. Describing them as legal ‘property’ rights, however, seems inapt. For one thing, patients exercise significant but not complete rights of excludability under this legislation. People or entities in possession of a patient’s personal information, including health information, are allowed to use it for a variety of purposes without the individual’s consent. Although it is possible for a patient to alienate personal health information (by, for example, allowing use), the patient may not be able to alienate the information totally, or totally unencumbered. The data often remains subject to the GDPR provisions exercisable by the patient. Finally, rights are not, strictly speaking, in rem. Patients can enforce their GDPR rights broadly, given the broad definition of ‘data processor’ (Art 4(7), 4(8)). Since some rights and obligations concern ‘data controllers’ only (not data processors or other persons), however, many of the rights are not good against ‘the world’—just the data controller, and data processors for whom the controller is responsible. Thus, on each of the three essential characteristics of property—excludability, alienability, and enforceability—rights under the GDPR are not property rights.

181
Herring, *supra* note 158, at 223.

182 Under the GDPR principle of transparency, data controllers are required to inform data subjects how they intend to process personal data, however this is generally conveyed in a Privacy Notice which provides a summary rather than case-by-case details: Arts. 13 and 14. See also Information Commissioner's Office, *Guide to the UK General Data Protection Regulation (UK GDPR), Right to be Informed*, available at https://ico.org.uk/for-organisations/guide-to-data-protection/guide-to-the-general-data-protection-regulation-gdpr/individual-rights/right-to-be-informed/ (accessed Dec. 8, 2020).

183 This is illustrated by the *Dinerstein* decision, in which it was held that the patient had not suffered any economic loss from the unauthorized use of his data (though it is worth noting that, had the court treated the information as property *per se,* a contractual claim for a ‘reasonable royalty’—applying only to use of property—might have been possible). *Dinerstein*, 484 F. Supp. 3d at 591–94.

184
*See*, *eg*, *White v Chief Constable of South Yorkshire Police* [1999] 2 AC 455; *Spartan Steel & Alloys Ltd v Martin* [1972] 3 WLR 502. ``Pure'' economic harm is similarly likely to be uncompensable in the United States. *Eg*, *532 Madison Ave. Gourmet Foods, Inc. v Finlandia Ctr., Inc.*, 276 A.D.2d 1016, 718 N.Y.S.2d 813 (2000).

185
*Wise v Kaye* [1962] 1 QB 639. In the U.S. emotional harm must usually be accompanied by some physical harm. But see, eg, Dillon v. Legg, 68 Cal. 2d 728, 441 P.2d 912 (1968) (stating elements of limited cause of action for negligent infliction of emotional distress); *Clinton v Jones*, 520 U.S. 681 (1997) (stating elements of cause of action for intentional infliction of emotional distress)*.*

186
Herring, *supra* note 158, at 224. Complaining to the Care Quality Commission or the General Medical Council, rather than a lawsuit, might be elicit disciplinary action by regulators for breaching a patient’s rights, but not compensation.

187
*See*, *eg*, *ABC v St Georges Hospital*, *supra* note 159. In the U.S., similar cases have arisen. In fact, the ABC court explicitly cited and quoted from U.S. caselaw. *Id.* (citing and quoting *Pate v Threlkel*, 661 So. 2d 278, 282 (Fla. 1995), Safer v. Estate of Pack, 677 A.2d 1188 (N.J. App. Div. 1996), and *Tarasoff v Regents of the University of California* 551 P.2d 334 (1976).

188 The case was remitted to the High Court for further evaluation of the facts. The High Court held that medical professionals owed a duty in the particular circumstances of this case to consider disclosing the patient’s confidential information to his daughter without his permission, but that the duty was not breached when they decided not to disclose: *ABC v St George’s Healthcare NHS Trust**supra* note 159.

189
*Oxford v Moss* (1979) 68 Cr. App. R. 183.

190
Herring, *supra* note 158, at 228.

191 A similar situation exists in the United States. See Computer Fraud and Abuse Act (CFAA) 18 U.S.C. § 1030 et seq. (making certain intentional access of computers without authorization or by exceeding authority a crime) See, eg, *LVRC Holdings LLC v Brekka*, 581 F.3d 1127, 1131 (9th Cir. 2009) (stating that section (g) of the CFAA makes it a crime to intentionally access a computer system without authorization or exceeding authorized access and thereby obtaining information from any protected computer resulting in an aggregate loss of $5,000 during a 1 year period.).

192 Acting in accordance with the patient’s permission is not entirely determinative. For example, a patient may authorise a health provider to share data with particular third-parties, but if the health provider is careless with data security when doing so, they might still be liable for negligence. The degree to which the patient was informed about the potential implications and risk is also relevant.

193 GDPR Art. 7; *Id.* Recital 32; Intersoft Consulting, *GDPR, Consent,*https://gdpr-info.eu/issues/consent/ (accessed Dec. 6 2019).

194 GDPR Art 4(11) (‘consent’ of the data subject means any freely given, specific, informed and unambiguous indication of the data subject’s wishes by which he or she, by a statement or by a clear affirmative action, signifies agreement to the processing of personal data relating to him or her’).

195
*Id.* Art. 9(2)(h), (i) and (j).

196
*Id*. Art. 9(2)(b), (d) and (f).

197 ‘Broad consent’ describes the consent given when a patient is provided with a general description of the types of research that may be conducted in the future, to which they consent.

198
*Id.* Art. 9(2)(h), (i).

199
Herring, *supra* note 158 at 232 (‘[M]any observers would say that, at ground level, the rights and duties arising from patient confidentiality are honored more in the breach than the observance.’ [citing Austen Garwood-Gowers, John Tingle, Tom Lewis, *Confidentiality, Access to Health Records and the Human Rights Act 1998, in*Healthcare Law: Impact of the Human Rights Act 1998 181–200 (2001).]).

200 Montgomery, *supra* note 38; de Witte & ten Have, *supra* note 24, at 51–55 (reviewing property theories of Locke, Bentham, Kant, and Nozick).

201 N. Purtova, *Property Rights in Personal Data: Learning from the American Discourse*, 25 Comput. L. & Secur. Rev. 507, 515 (2009) (citing Murphy, *Property Rights in Personal Information: An Economic Defense of Privacy* 83 Geo. L.J. 2381 (1996) at 2383–84); Purtova, *supra* note 38, at 83–84, 86.

202
*For example*, Determann, *supra* note 16 (arguing that there should be no property rights in data).

203 Patrick Hummel, Matthias Braun, Peter Dabrock, *Own Data? Ethical Reflections on Data Ownership, in*Philosophy andTechnology (2020); Katherine A. Mikk, Harry A. Sleeper, Eric J Topol, *The Pathway to Patient Data Ownership and Better Health,* 318(15) JAMA 1433 (2018); Roberts, *supra* note 10; McGuire, Roberts, Aas, & Evans, *supra* note 24, at 62. James Rule & Lawrence Hunter, *Towards Property Rights in Personal Data*, *in*Visions of privacy: policy choices for the digital age 168–181 (Rebecca A. Grant & Colin J. Bennett eds., 1999); Purtova, *supra* note 241. For media coverage: FT.com, *Digital Privacy Rights Require Data Ownership*, FinancialTimes (2018), https://www.ft.com/content/a00ecf9e-2d03-11e8-a34a-7e7563b0b0f4 (accessed Feb. 14, 2020).

204
Graeme Laurie, GeneticPrivacy: A Challenge toMedico-LegalNorms (2002); Graeme Laurie, *Privacy and Property? Multi-level Strategies for Protecting Personal Interests in Genetic Material* (2003), available at https://www.researchgate.net/publication/277049351_Privacy_and_Property_Multi-level_Strategies_for_Protecting_Personal_Interests_in_Genetic_Material (last accessed June 12, 2021). [Hereinafter Laurie, *Privacy and Property?*].

205 Human Rights Act 1998 (UK) First Protocol, Article 1.

206 I. Glenn Cohen, *Is There a Duty to Share Healthcare Data? in* I. GlennCohen, HollyFernandezLynch, EffyVayena, UrsGasser (eds), BigData, HealthLaw, andBioethics 214–15 (2018).

207 Guido Calabresi and A Douglas Melamed, *Property Rules, Liability Rules, and Inalienability: One View of the Cathedral*, 85 Harv. L. Rev. 1089 (1972). Contreras argues for this approach with respect to genetic information. Jorge L. Contreras, *Genetic Property*, 105 Geo. L.J. 1 (2016) [hereinafter Contreras, *Genetic Property*].

208 Laurie, *Privacy and Property? supra* note 204.

209 In the U.S. there is no generally applicable, federal legislation for the protection of personal data or privacy. The Health Insurance Portability and Accountability Act (‘HIPAA’) is a sector specific approach. Landmark State legislation, the California Consumer Protection Act, is broader in some senses, but narrower in others. It is a consumer protection statute that governs only large businesses and those holding large amounts of consumer data relating to the residents of one state. Sensitive data, including health data, can be transferred and sold to third parties for any purpose unless consumers affirmatively ‘opt-out’: Laura Bradford, Matteo Aboy & Kathleen Liddell, *International Transfers of Health Data Between the EU and U.S.: A better Approach for the U.S. to Ensure an `Adequate' Level of Protection*, J. of L. & Biosciences, 1, 24 (2020).

210
*See*, *eg*, Purtova, *supra* note 241, at 514–518 (reviewing U.S. perspectives); V. Bergelson, *It’s Personal, but is it Mine? Toward Property Rights in Personal Information*, 37 UC Davis L. Rev. 379 (2003). But see McGuire, Roberts, Aas, & Evans, *supra* note 24, at 67. For non-U.S. perspective on why we cannot avoid thinking in terms of property, see Purtova, *supra* note 38.

211 Health Insurance Portability and Accountability Act of 1996, (42 U.S.C. § 1320d et seq.) (defining covered entities to include health plans, health care clearinghouses, health care providers that transmits health information in electronic form). The HIPAA Privacy Rule, first adopted in 2000, is codified at 45 C.F.R. § 160–164. 65 Fed. Reg. 82462, Dec. 28, 2000.

212 Bradford *et al*., *supra* note 209, at 21–22, 27–28. HIPAA’s reach is narrower than the GDPR but it provides strict, GDPR-like rules for ‘protected health information’. This is defined to include any health-related information that can be used to identify a particular individual. Under HIPAA, as under the GDPR, use or disclosure of personal information is forbidden unless the subject explicitly consents or a specific exception applies. Covered entities may freely use and disclose personal information without prior permission for treatment, payment, operations and certain public benefit activities such as research or law enforcement activities. These exceptions are similar to GDPR’s list of lawful bases for processing such as vital interests of the subject, performance of a contract, a task carried out in the public interest or the legitimate interests of the processor. Both laws require additional disclosures and safeguards before individual data can be used for ‘marketing’ purposes.

213 Bradford *et al*., *supra* note 209; B. Evans, *Much Ado About Data Ownership*, 25 Harv. J. L. & Tech. 69, 82 (2011).

214 HIPAA, *supra* note 211.

215 Bradford *et al*., *supra* note 209, at 28–29. The HIPAA Privacy Rule contains numerous exceptions when authorization for disclosure is not required. 45 C.F.R. § 164.512.

216 Wolf et al, *supra* note 141, at 6–9 (explaining three risks faced by research participants: participant information may be used for objection purposes, may be inadvertently or involuntarily disclosed, may be used against the participant); Clayton, Evans, Hazel, & Rothstein, *supra* note 126, at 4 (explaining the balancing between individual concerns and public access, and noting that ‘The tradeoffs often implicate both personal and societal interests, which vary depending on the context.’). This becomes clear once one considers that HIPAA is not the only statute in play. A recent comprehensive review of U.S. state and federal laws revealed a ‘web of legal protections’ governing *genetic* information of research participants. *Id.* at 7–9, 45–53, 58 (discussing HIPAA and state laws governing privacy of personal health information). *Id.* at. 61–62 (explaining that state laws may offer models to close the gaps in federal laws concerning genetic information of research participants). *See* Clayton, Evans, Hazel, & Rothstein, *supra* note 126, at 32.

217 For example J. Litman, *Information Privacy/Information Property’*, 52 Stan. L. Rev. 1283, 1296–1297, 1300–1301 (2009); P.M. Schwartz, *Property, Privacy, and Personal Data*, 117 Harv. L. Rev. 2096, 2096–2097 (2003).

218 Property rights do not restrain downstream uses if patient consents to assign or transfer property rights downstream.

219 This is also view also put forward in Evans, *supra* note 213.

220 Laurie, *Privacy and Property?*, *supra* note 204, at 10. Ironically, Professor Laurie thinks that property may actually serve a more meaningful purpose here—because it allows for residual rights of control of the kind described by Professor Hall, below.

221 Mark A. Hall, *Property, Privacy, and the Pursuit of Interconnected Electronic Medical Records*, 95 Iowa L. Rev. 631, 660 (2010).

222
*Id.* *See also* M.A. Hall & K. A. Schulman, *Ownership of Medical Information*, 301 JAMA 1282–1284 (2009) (arguing that certain ownership stakes, licensing agreements, etc. in patient data may provide incentives to create beneficial uses of information).

223
*Id.* at 661.‘In practice, [the proposal] would permit the transfer for an initial category of use of personal data, but only if the customer is granted an opportunity to block further transfer or use by unaffiliated entities. Any further use or transfer would require the customer to opt in—that is, it would be prohibited unless the customer affirmatively agrees to it.’; Schwartz, *supra* note 217, at 2098.

224 Julie E. Cohen, *Examined Lives: Informational Privacy and the Subject as Object*, 52 Stan. L. Rev, 52, 1373 (2000). *See* Sonia M. Suter, *Disentangling Privacy from Property: Toward a Deeper Understanding of Genetic Privacy*, 72 Geo. Wash. L. Rev. 737 (2004) (arguing that property cannot provide an adequate framework for protecting privacy in genetic information because property commodifies and privacy is fundamentally concerned with trust-based relationships not subject to commodification).

225 Stepanov, *supra* note 41. *See eg*, Richard A. Posner, Economic analysis of law 31–91 (6th edn 2003); Richard A. Epstein, *A Clear View of The Cathedral: The Dominance of Property Rules*, 106 Yale L. J. 2091 (1997).

226 Henry E. Smith, *Intellectual Property as Property: Delineating Entitlements in Information*, 116 Yale L. J. 1742 (2007).

227 Hall and Schulman, *supra* note 222, at 1283; *See also* E. Haislmaier, *Health Care Information Technology: Getting the Policy Right*, TheHeritageFoundation (2006).

228 For economists, property rights are a vehicle used to help solve this problem, which arises in the context of so-called ‘public goods.’ See *infra* Part V.E., *Robert Cooter & Thomas Ulen*, Law & Economics 40 (2016).

229 Schwartz, *supra* note 217, at 2097.

230 Schwartz, for example, argues that private ownership of information could be achieved in a ‘limited’ (ie circumscribed) way, like IP rights. *Id.* at 2096–2010.

231 The definition of exigible, as stated in the English Oxford dictionary, is: ‘able to be charged or levied, able to be demanded or exacted.’ OxfordEnglishDictionary, https://en.oxforddictionaries.com/definition/exigible (accessed Dec. 6, 2019).

232 Hall, *supra* note 221, at 649; M. A. Heller, *Can Patents Deter Innovation? The Anticommons in Biomedical Research*, 280 Science 698–701 (1998) The dynamic has been termed the ‘tragedy of the anticommons’ to show that there are problems with private ownership of non-rivalrous things (underutilization) as well as common ownership of rivalrous things (over utilization). The latter has long been known as the ‘tragedy of the commons’.

233 Mirchev, *supra* note 14.

234 Rodwin, *supra* note 38. (Arguing that the public ownership model will eliminate the anti-commons problem by opening data up to experimentation and use by multiple parties at the same time). Rodwin, *supra* note 38; Hall, *supra* note 38; See also Marshall Van Alstyne, Erik Brynjolfsson & Stuart Madnick, *Why Not One Big database? Principles for Data Ownership*, 15 Decis. SupportSyst. 267–284 (1995).

235 Montgomery, *supra* note 38, at 83.

236 Rodwin, *supra* note 38, at 86–88.

237 Hall, *supra* note 42 220, 647–648.

238 In economics the term ‘network effects’ or ‘natural monopoly’ ‘exists when average costs fall as the scale of production rises.’ Cooter and Ulen, *supra* note 228, at 127. As more and more individuals use the product or service, the production (use) of the service increases but the cost of producing the good (ie, the information) goes down. *See*YochaiBenkler, The Wealth of Networks: How Social Production Transforms Markets and Freedom (2006). One reason this occurs is because the cost of producing the information does not rise as it is produced. Another reason this occurs is because the cost of production will actually decrease as more information is produced. If data is gathered by a central authority such as the NHS, and that central authority invests in proper infrastructure, the cost to maintain and service the infrastructure will decrease over time, while the production of information will increase.

239 Knapton, *supra* note 12.

240 Powles and Hodson, *supra* note 6.

241 The Clinical Practice Research Datalink allegedly licensed the use of anonymized patient data from NHS GP surgeries to large pharmaceutical corporations, and the Department of Health provided technical medical information at no cost to Amazon in order to use in its voice assistants: Toby Helm, *Patient Data from GP Surgeries Sold to U.S. Companies*, TheGuardian, 2019, https://www.theguardian.com/politics/2019/dec/07/nhs-medical-data-sales-american-pharma-lack-transparency (accessed Feb. 14, 2020); Toby Helm, *Revealed: How Drugs Giants Can Access Your Health Records*, TheObserver, 2020, https://www.theguardian.com/technology/2020/feb/08/fears-over-sale-anonymous-nhs-patient-data (accessed Mar. 2, 2020); Confidentiality Advisory Group, *Minutes of the Meeting of the Confidentiality Advisory Group* 2–5 (2018); Shaun Lintern, *Conservative Government Giving NHS Data to Amazon for Free, Documents Reveal*, TheIndependent, 2019, https://www.independent.co.uk/news/health/amazon-nhs-data-access-uk-government-contract-a9237901.html (accessed Feb. 14, 2020).

242 Evans, *supra* note 24, at 103–104.

243 Litman, *supra* note 217, at 1294–98.

244
*See* Section III.B.

245 Litman, *supra* note 217, at 1294–98.

246
*See*, *eg*, Stuart Mills, *Who Owns the Future? Data Trusts, Data Commons, and the Future of Data Ownership*, SSRN Journal (2019); Delacroix and Lawrence, *supra* note 29; JeremiahWau, JamesPenner & BenjaminWong, *The Basics of Private and Public Data Trusts* (2019), http://law.nus.edu.sg/wps (accessed Feb. 14, 2020).

247 Wendy Hall & Jerome Pesenti, *Growing the Artificial Intelligence Industry in the UK* (2017).

248 Sean McDonald, *Reclaiming Data Trusts | Centre for International Governance Innovation* (2020), https://www.cigionline.org/articles/reclaiming-data-trusts (accessed Feb. 21, 2020).

249 Keith Porcaro, *In Trust, Data*, 2019 Minn. L. Rev. Headnotes 332.

250 Delacroix and Lawrence, *supra* note 29.

251 McDonald, *supra* note 248.

252 Christopher Reed, BPE Solicitors, and Pinsent Masons, *supra* note 28, at 14, 17, 21; Hall and Pesenti, *supra* note 247, at 46; Delacroix and Lawrence, *supra* note 29. However some authors argue that property in data is not a necessary feature of a data ‘trust’; Jeremiah Lau Jia Jun, J.E. Penner, Benjamin Wong, *The Basics of Private and Public Data Trusts*, NUS Law Working Paper 2019/019 (Sept. 2019), available at https://papers.ssrn.com/sol3/papers.cfm?abstract_id=3458192.

253 An exception is the article by Delacroix and Lawrence, *supra* note 29. They argue that ‘rights’, as well as property, can be the subject of legal trusts (citing McFarlane and Mitchell) (section 2.4.1). Hence data trusts could be set up as formal ‘legal trusts’ rather than contractual or corporate frameworks (section 2.4.3). Notably, even Delacroix and Lawrence, take the view that a legal ‘data trust’ does not hinge on data being property, but rather on the possibility of assigning rights of portability, access and erasure p 20–21 (last sentence of section 2.3).

254 Rinik notes the language of ‘data trust’ is essentially a nomenclature intended to inspire trust and confidence. Christine Rinik, *Data Trusts: More Data Than Trust? The Perspective of the Data Subject in the Face of a Growing Problem*, 34 Int'l Rev. L., Computers, & Tech 342–363 (2019). An alternative, more accurate, less directional term could be ‘data warehouse’ or ‘data clearinghouse’. Delacroix and Lawrence, *supra* note 29, argue that ‘rights’, as well as property, can be the subject of legal trusts (citing McFarlane and Mitchell) (section 2.4.1).

255 European Commission, *Proposal for a Regulation of the European Parliament and of the Council on European Data Governance (Data Governance Act*) 2020/0340 (COD), see especially Art 4.

256 WiseGuy Reports, *supra* note 3.

257
*See generally* Purtova, *supra* note 38.

258 Hall, *supra* note 38, at 642–643.

259 Hummel et al, *supra* note 203; Roberts, *supra* note 203; Schwartz, *supra* note 217.

260
*See* *supra* Section IV.

261 Litman, *supra* note 217 at 1294–1298; Evans, *supra* note 255 at 103–104; Petere Swire, *Financial Privacy and the Theory of High-Tech Government Surveillance*, 77 Wash. Univ. L.Q. 461 (1999).

262 Herring, *supra* note 158, at 196–197; JonathanHerring, Q&A Medical law 1, 104 (2016).

263
*See* Section IV.

264 See *supra* Section IV. Evans notes that the current safety valves under the Health Insurance Privacy and Protection Act (HIPPA) in the U.S. ‘offer a framework of patient entitlements and protections strikingly similar to what patients would enjoy if they owned their data.’ Evans, *supra* note 24, at 82. She also notes that even a property framework would not guarantee more protection of patient privacy/information because one must still specify what kind of property rights patients would have in their health information. *Id.* at 77–81.

265 Professor Contreras makes a related argument in the context of genetic information. Contreras, *Genetic Property*, *supra* note 207. (Arguing for regulation of genetic property that makes most research uses of genetic information presumptively permissible and subjects violators to liability rules rather than property rules); Jorge L. Contreras & Francisca Nordfalk, *Liability (and) Rules for Health Information*, 29 HealthMatrix 179 (2019) (arguing for a regulatory regime to govern the use of private health information that does not require consent for ‘information derived from physical samples’ and private rights of action to enforce violations).

266 Pamela Samuelson, *Privacy as Intellectual Property?* 52 Stan. L. Rev. 1125 (2000)1140–1141; Mark A. Lemley, *Private Property*, 52 Stan. L. Rev. 1545, 1550–1552 (2000); Marc A. Rodwin, *Patient Data: Property, Privacy & the Public Interest*, 36 Am. J. L. & Med. 586, 559–601 (2010).

267
Cooter and Ulen, *supra* note 228.

268
*Id.*

269
*Id.*

270
*Id.*

271 Samuelson, *supra* note 266, at 1140–1141; Lemley, *supra* note 266, at 1550–1552.

272 Evans, *supra* note 213, at 75–77.

273 Stephen Armstrong, *Data, Data Everywhere: the Challenges of Personalized Medicine*, BMJ j4546, 2 (2017).

274
*See, eg*, the proposal for a Common health data space: European Commission, *Communication from the Commission to the European Parliament, The Council, The European Economic Social Committee and the Committee of the Regions: A European strategy for data* COM/2020/66 final. See also the workstreams of the Global Alliance for Genomics and Health (GA4GH), which include data security standards, regulatory rules on data sharing, standard ontologies for clinical and phenotypic data capture, standards for cloud-based task and workflow execution, harmonisation of data use and researcher identities, standards-based components for downstream analysis, available at https://www.ga4gh.org/how-we-work/workstreams/ (accessed Feb. 19, 2020). In the USA, see Ctrs. Medicare & Medicaid, PromotingInteroperabilityPrograms, https://www.cms.gov/Regulations-and-Guidance/Legislation/EHRIncentivePrograms (accessed Nov. 11, 2020).

275 Evans, *supra* note 213, at 86–106.

276 One of us has argued that better information requires government subsidies or private incentives, but not property. Simon, *Off-Label Innovation*, *supra* note 102.

277 Ritter and Mayer, *supra* note 5, at 221–223, 226–227.

278 We leave open the question whether other scholars’ approaches are appropriate or preferred. *E.g.*, Contreras & Nordfalk, *supra* note 264.

279
*See eg,* Data Protection Act 2018 (UK), s. 188.

280 Annie Sorbie, Wifak Gueddana, Graeme Laurie, David Townend, *Examining the Power of the Social Imaginary Through Competing Narratives of Data Ownership in Health Research*, 68 J. of L. & Bioscis. 1, 5–6 (2021); Jacqueline Lipton, *Information Property: Rights and Responsibilities*, 56 Fl. L. Rev. 135, 141 (2004) McGuire, Roberts, Aas, & Evans, *supra* note 24, at 64.

281
*Id.*

